# Stability and compatibility of resorcin[4]arene hexamer cages with amphiphilic random copolymers in organic solvents

**DOI:** 10.1039/d6ra01475e

**Published:** 2026-05-11

**Authors:** Marah Alqaisi, Wolfgang H. Binder

**Affiliations:** a Macromolecular Chemistry, Institute of Chemistry, Faculty of Natural Science II (Chemistry, Physics and Mathematics), Martin-Luther-University Halle-Wittenberg von-Danckelmann-Platz 4 Halle D-06120 Germany

## Abstract

The structural integrity and guest–hosting capabilities of resorcin[4]arene-based hexamer cages ([Rs_4_]_6_ and [Rs_11_]_6_) bearing different side chains (–C_4_H_9_, butyl or –C_11_H_23_, undecyl) is explored using water-soluble copolymers mapped in different solvents (CHCl_3_, CH_2_Cl_2_, toluene and THF). We here identify significant differences between the length of the macrocycle's “footing” chains (–C_4_H_9_ or –C_11_H_23_) and their intrinsic stability. NMR and DLS size analyses reveal that while both cages are stable in chloroform, the undecyl-footed [Rs_11_]_6_ cage demonstrates superior intrinsic stability in toluene and dichloromethane, self-assembling into discrete hexamers (*D*_h_ = 3.6 nm) even in the absence of embedded guests. In contrast, the butyl-footed [Rs_4_]_6_ cage requires an alkylammonium salt template to avoid formation of large and unstable aggregates. When mixed with amphiphilic random copolymers, the cage [Rs_11_]_6_ maintains high encapsulation efficiencies at molar ratios of up to 1 : 6 (cage : copolymer), regardless of the copolymer's philicity, displaying either aliphatic or fluorinated sidechains. A competitive interaction between the copolymers' polyethylene glycol (PEG) side chains and structural water molecules is proposed, establishing the cage [Rs_11_]_6_ as a robust and versatile scaffold for the development of complex, water-soluble nano-assemblies. This now offers a resilient platform for biomimetic carrier and catalytic applications.

## Abstract 2

Structural integrity of resorcin[4]arene-based hexamer cages with different side chains (–C_4_H_9_, butyl or –C_11_H_23_, undecyl) is achieved with water-soluble copolymers in different solvents at molar ratios of up to 1 : 6 (cage : copolymer).

## Graphical abstract

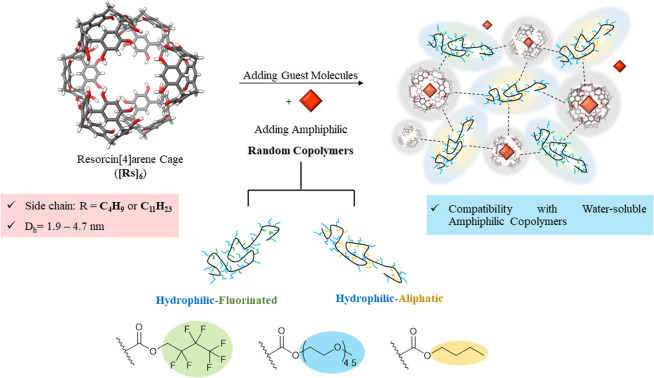

## Introduction

**Fig. 1:**
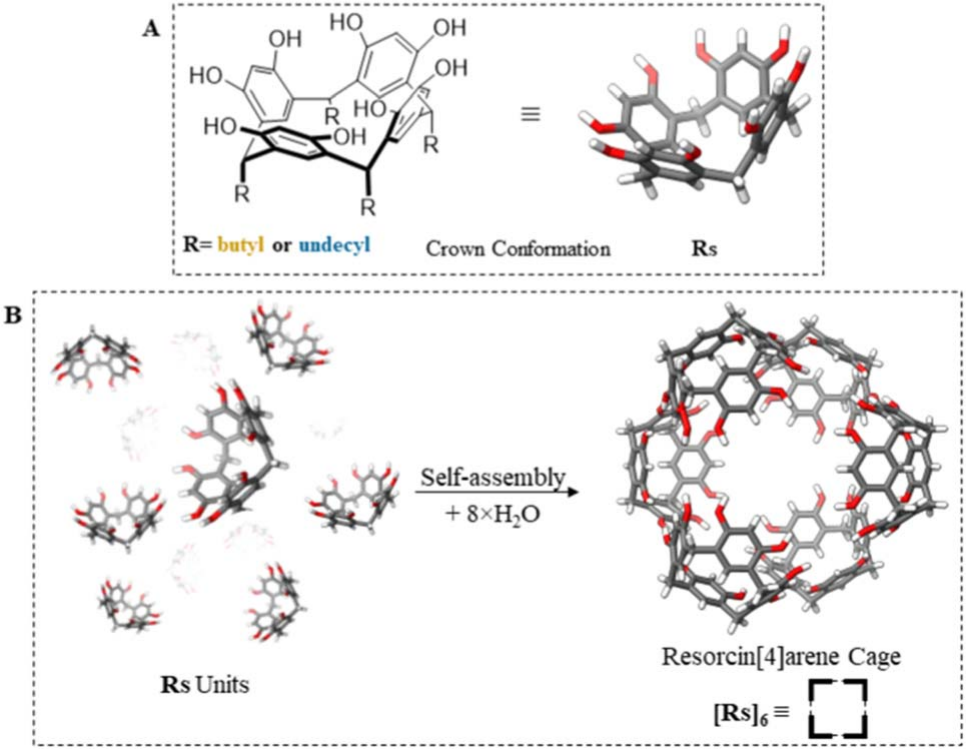
Resorcin[4]arene units (Rs) with crown conformation (A) form resorcin[4]arene cages [Rs]_6_ with different footing side chains in organic solvents with 8 water molecules (B).

**Fig. 2:**
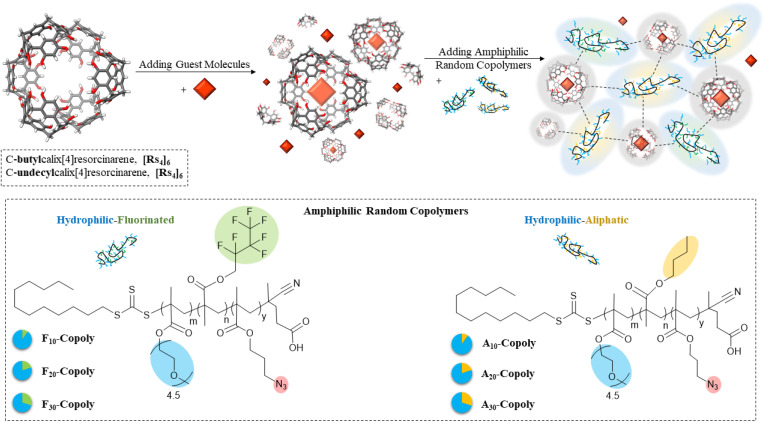
Interacting amphiphilic random copolymers with resorcin[4]arene cages bearing different footing sidechains (*C*-butylcalix[4]resorcinarene, [Rs_4_]_6_ and *C*-undecylcalix[4]resorcinarene, [Rs_11_]_6_) in the presence of chromophore guest to investigate the stability of the cage formation and its functionality. Each random copolymer displays two different affinities and produced in three different molar compositions; from 10 mol% to 30 mol% of the hydrophobic component. The crosslinking azide-moieties are embedded into the hydrophobic part. Blue-shaded side chains are hydrophilic, yellow-shaded side chains are aliphatic and green-shaded side chains are fluorinated.

Self-assembled structures in nature have always inspired research studies to mimic their functionalities and characteristics.^1^ Supramolecular organic cages with confined nano-spaces have always been a topic of interest since they offer properties that the bulk phase does not provide. As a prominent example the formation of compartments inside a cage can stabilize the transition state of a chemical reaction, so exerting a catalytic function,^2^ or generate synthetic biomimetic or ‘life-like’ supramolecular structures that resemble cellular and sub-cellular organisms.^3–6^ Recently, selective catalytic permeability and active transport functionalities have been probed by resorcin[4]arenes cages. Their selectivity and hosting ability towards various guest molecules make them excellent candidates for enhancing functionality and biomimicry of transporter vesicles or liposomes carrying specific molecules to targeted biological systems.^7–9^ Resorcin[4]arene units (Fig. 1A) (abbreviated as Rs) can noncovalently self-assemble to form discrete and well-defined cages (Fig. 1B). A single cage is made from 6 crown-shaped macrocycles resorcin[4]arenes and 8 water molecules to form a hexamer cage through 60 hydrogen bonds as first reported by MacGillivary and Atwood in 1997.^10^ The cage resembles an inflated cube with one unit at each side and a water molecule positioned at each of its corners.^11,12^ A resorcin[4]arene unit is easily prepared *via* condensation between resorcinols and aldehydes to produce a macrocycle composed of 4 resorcinol units.^13^ The delocalization of the negative charge across the hexamer structure makes it acidic (p*K*_a_ = 5.5), so acting as a catalyst as reported by Tiefenbacher and coworkers.^14^ They reported a successful tail-to-head cyclization of terpenes, mimicking cyclase enzymes.^15–17^ The encapsulation behaviour of this cage arises from its π- and hydrogen bonding interactions. When hosting ionic species, cations are stabilized by π orbitals of the aromatic rings (cation–π interactions), and anions are neutralized through hydrogen bonds. The cages can host polar guests through its hydrogen bonds, like carboxylic acids, amino acids and alcohols.^18–20^ By offering a restricted space of 1400 Å^3^, it is imposing a steric hindrance on the substrate and the guest catalyst, which makes it selective for the substrate and the product. Bianchini *et. al.* reported catalytic hydration of neutral isonitriles to yield *N*-formylamides by reversible encapsulation of the guest molecule.^21^ La Sollera *et. al.* demonstrated the catalytic activity of the cage to catalyse 1,3-cycloaddition reaction between diazoacetate esters and electron poor alkenes.^22^ Catti *et*. *al*. explored the cage's ability to perform hydroalkoxylation of unsaturated alcohols to cyclic ethers under mild conditions.^23^ Caneva *et al.* reported the isomerization reaction of epoxides to their corresponding carbonyl compounds within the cage's internal space.^24^ Interestingly, Cavarzan *et. al.* reported unprecedented encapsulation of Au-catalysts and its substrate within the cage's cavity. The imposed steric-hindrance effect on the complex yielded unexpected products (Fig. 2).^25^

The self-assembly and stability of resorcin[4]arene hexamer cages is highly sensitive to environmental factors, as studied by Rebek and coworkers,^26–28^ guided *via* hydrogen-bonds in non-polar solvents, provided that a small amount of water molecules is present.^29^ As the assembled cage is in dynamic equilibrium with its monomeric resorcin[4]arene units, its stability depends on the nature of solvent, the addition of guest molecules, the concentration of mixing, temperature and the presence of polar additives to induce cage formation.^27^ The necessity of water molecules was confirmed by the observation that anhydrous organic solvents, such as dichloromethane, yielded no cage, proving that water acts as a crucial hydrogen-bonding template for the hexamer assembly in nonpolar media. In addition, polar solvents, such as methanol, can disrupt the intra-cage hydrogen bond network, so disassembling the cage.^27^

Alkylammonium salts are among the most exploited guests for studying the reversible capture and release mechanism involving solvent exchange, due to cation–π interactions and the characteristic ^1^H-NMR resonances upon encapsulation. The hydrophobic nature of their alkyl chains matches the cage's internal hydrophobic cavity, further enhancing host–guest interaction, so that encapsulation is also dictated by size.^19,28,30^ Furthermore, metal-complexes are another important example of stabilizing guests. Thus recent studies have demonstrated the versatility of ruthenium-based complexes as guests within resorcin[4]arene cages, serving both structural and catalytic purposes. Beyeh *et. al.* utilized the bulky, pseudooctahedral ([Ru(bpy)_3_]^2+^) cation to enhance the structural integrity of the cage. ESI-ToF mass spectrometry indicated that its significant volume and templating effect provides superior stabilization compared to standard ammonium-based guests.^12^ Hkiri *et. al.* successfully encapsulated a neutral ruthenium catalyst within these cages, confirming the guest's insertion through DOSY-NMR spectral analysis by observing matched diffusion coefficients. This encapsulated system was then employed for the oxidation of arylmethyl alcohols, where the physical constraints of the cage's interior provided substrate selectivity that was otherwise absent in bulk phase reactions.^31^ Furthermore, the electronic environment of the cage influences the guest's photophysical properties, as seen in Ichou *et. al.* work where the encapsulation of a cationic Cu(ii) complex resulted in a hypsochromic (blue) shift of its d–d absorption bands.^32^

Resorcin[4]arene-based complex structures have been studied for their ability to mimic cell membrane proteins, acting as selective receptors, channels, and transporters.^7–9,33–35^ Muthwill *et. al.* explored the hexamer cage within a polymeric membrane built from a triblock copolymer of poly(2-methyl-2-oxazoline)_7_-*block*-poly(dimethylsiloxane)_50_-*block*-poly(2-methyl-2-oxazoline)_7_ (PMOXA_7_-*b*-PDMS_50_-*b*-PMOXA_7_) to achieve selective transport.^34^ However, the stability and interaction profile of the cage with the used copolymers remains largely unclear. Critical seems to be the hydrophobic/hydrophilic balance within these copolymers, with a clear pattern of stabilizing/destabilizing the cage remaining unknown.

We here aim to develop an interaction-profile between resorcin[4]arene cages and surrounding copolymers, to further unlock the potential of resorcin[4]arene cages for next-generation polymeric nanostructures with nanosized compartments, then projected to interact with the surrounding polymers. Hence, we have used two cages with different lengths of their ‘footing’ side chains in an attempt to tune the cages' interactions with the surrounding co-polymer to integrate the cages with novel amphiphilic random copolymers -previously reported by us^36^ (Fig. 1). We focus to investigate the effects of the copolymers on both cages, displaying different hydrophobic/hydrophilic profiles, exemplified by their aliphatic-(A) and fluorinated- (F) side chains, ranging from 10 mol% (A_10_, F_10_) up to 30 mol% (A/F_30_-Copoly). Poly(ethylene glycol) side chains (PEG, *n* = 4.5) are used as the hydrophilic part inside the random copolymers. Absorption and emission spectroscopy were employed to characterize encapsulation of a chromophore metal-complex guest to verify the cage's integrity and selective confinement in the presence of the respective copolymers. We also mapped the stability of the two resorcin[4]arene cages in different solvents to probe the effects that the guest or solvent might have on both cages. Analytical techniques, including 1D ^1^H- and DOSY-NMR spectroscopy and dynamic light scattering (DLS) were used for assessing long-term stability by the hydrodynamic diameter, in presence and absence of ammonium-based guest molecules. The outcome is directed to generate cages with the potential of being embedded into a more complex polymeric systems, thereby opening new avenues in synthetic biomimetic systems.

## Experimental

### Materials

Resorcinol (99%, Roth), dodecanal (95%, BLDpharm), pentanal (98.5%, Sigma-Aldrich), hydrochloric acid (37 w%, Grüssing), tetrahexylammonium bromide (THA-Br) (99%, Sigma-Aldrich), tetraoctylammonium bromide (TOA-Br) (Fluka), tetrabutylammonium bromide (TBA-Br) (>99%, TCI acetone-*d*_6_ (99.8%, Chemotrade), tris(4,4′-dimethyl-2,2′-bipyridine)ruthenium bis(hexafluorophosphate) (Ru(dmbpy)_3_(PF_6_)_2_) (95%, Sigma-Aldrich), chloroform-d_3_ (CDCl_3_) (stabilized with Ag, ≤100%, ARMAR), toluene-d_8_ (99.8%, ARMAR), dichloromethane-d_2_ (95.5%, ARMAR), ethanol (99.8%, Fisher Chemical). We used freshlydistilled solvents: chloroform (CHCl_3_), dichloromethane (DCM), tetrahydrofuran (THF), toluene, methanol, de-ionized water (from TKA-GenPure system).

### Characterization methods

^1^H- and ^13^C-NMR spectra were recorded with Agilent Technologies 400 MHz VNMRS and Agilent Technologies 600 MHz shielded VNMRS spectrometers at 27 °C in CDCl_3_, toluene-d_8_, DCM-d_2_ and acetone-d_6_. MestReNova (14.2.1-27684) was used as a software to analyse the spectra. ^1^H-DOSY NMR was recorded by using Agilent Technologies 400 MHz VNMRS spectrometer at 27 °C the previously mentioned deuterated solvents with 1.0 ms – 3.0 ms as gradient time and 100 ms – 300 ms as diffusion time. MestReNova (14.2.1-27684) was used as a software to analyse the spectra. Dynamic light scattering (DLS) measurements were done using Litesizer 500 from Anton Paar with 10 mm low volume quarts cuvette. The irradiation wavelength was 658 nm with detection angles at 90° (side scattering) or at 175° (back scattering) or at 15° (forward scattering). The temperature was set to 25 °C with varying sample concentrations in organic solvents. Solvents and samples were filtered (pore size: 0.2–0.4 µm) prior preparation and measurements. ESI-TOF-MS measurements were done with Bruker Daltonics microTOF *via* direct injection at a flow rate of 180 µL h^−1^ in positive and negative modes with an acceleration voltage of 4.5 kV. Samples were prepared by dissolving in methanol or in acetone or in chloroform or in acetone/chloroform (1 : 2) (0.1–0.3 mg mL^−1^), depending on the preparation methods as mentioned in subsequent sections. The instrument was calibrated using the ESI-L low concentration tuning mix from Agilent Technologies (product no. G1969-85000). The software Data Analysis (version 4.0) was used for evaluation. UV/vis-absorption measurements were done with PerkinElmer LAMBDA 365 UV/vis Spectrophotometer using Helma analytics quartz glass cuvettes with diameter 10 mm and screw cap. Ru-complex (Ru(dmbpy)_3_(PF_6_)_2_), the guest concentration was always 10 µM, resorcin[4]arenes at 60 µM (Ru-complex/resorcin[4]arenes: 1 : 6) with varying copolymers concentrations (resorcin[4]arenes/copolymers: 9 : 1 or 1 : 1). The cage–guest system was incubated overnight at 45 °C and sonicated ∼30 minutes before sample preparation. The samples were incubated overnight at 45 °C before measurements. Samples size: 3 ml. The temperature was controlled and set at 25 °C with waiting time of 3–5 minutes before measurements. Emission spectra were measured on a Cary Eclipse fluorescence spectrometer of Agilent for the same samples used for UV/vis spectroscopy with the same conditions. The acquired data was processed using OriginPro 2029 software. 3D structures of resorcin[4]arene unit and cage were drawn using UCSF ChimeraX version 1.10rc202505100155 (2025-05-10). https://www.rbvi.ucsf.edu/chimerax. The atomic distances were adapted from the ref. 37.

### Synthesis of resorcin[4]arene units (Rs_4_ and Rs_11_)

The method was adopted and modified from previous work.^13,38^ Hydrochloric acid (37w%, 1 mol equivalent) was added to ethanol (99.8%, *c* ≈ 4 M) and stirred for few minutes. Resorcinol (1 mol equivalent) was added and the solution was brought to −1.7 to −1.5 °C. Dodecyl aldehyde for Rs_11_ or pentanal for Rs_4_ (1 mol equivalent) was dissolved in ethanol and added drop-wise over 90 minutes using dropping funnel. The temperature was kept below 0 °C and the solution was stirred through the addition process. After addition of the aldehyde, the solution was brought to room temperature and placed in an oil bath. The temperature of the oil bath was increased until 100 °C. The reaction was heated to reflux for approximately 18 hours. After stopping by cooling to room temperature the product started precipitating as a yellow powder. The crude product was dispersed in cold methanol (−10 °C) and filtered. The procedure was repeated 3 times until the supernatant was clear and washed excessively with deionized water until the solution indicated neutral pH level. The yellowish powder was crystallized in methanol (at approximately 54 °C) and cooled to room temperature. It was then filtered with a solution of cold methanol/water (v/v, 1 : 1) and then cold methanol (∼4 °C). The slight yellowish powder was dried at 45 °C at 90 mbar for 2 hours. The product was collected in a container that is open to air to keep it moisturized. The identity was confirmed using ESI-ToF (Fig. S43 and S44) and ^1^H- and ^13^C- NMR (Fig. S10) in acetone-*d*_6_.

Rs_11_: *M* = 1105.68 g mol^−1^. ^1^H-NMR (402 MHz, acetone-*d*_6_, *δ* in ppm): 8.41 (s, 6H), 7.52 (s, 3H), 6.22 (s, 4H), 4.29 (s, 4H), 2.77 (s, 3H), 2.29 (s, 8H), 1.29 (s, 75H), 0.88 (s, 12H).

^13^C-NMR (101 MHz, acetone-*d*_6_, *δ* in ppm): 205.17, 151.79, 124.48, 124.29, 102.78, 33.48, 33.42, 31.77, 29.69, 29.60, 29.58, 29.54, 29.47, 29.34, 29.28, 29.23, 29.14, 29.09, 28.95, 28.89, 28.76, 28.71, 22.44, 13.46.

Rs_4_: *M* = 712.92 g mol^−1^. ^1^H-NMR (402 MHz, acetone-*d*_6_, *δ* in ppm): 8.41 (s, 7H), 7.56 (s, 4H), 6.24 (s, 4H), 4.30 (s, 4H), 2.29 (s, 8H), 1.38 (s, 8H), 1.28 (s, 9H), 0.89 (s, 13H).

^13^C-NMR (101 MHz, acetone-*d*_6_, *δ* in ppm): 206.12, 152.72, 125.49, 125.29, 103.72, 34.33, 34.06, 31.31, 29.84, 23.37, 14.56.

### Preparation of cage–guest samples for absorption and emission spectroscopy in chloroform

Stock solutions in CHCl_3_ were used for all sample preparations and prepared with the following concentrations: (a) [Ru(dmbpy)_3_]^2+^[(PF_6_)_2_]^2−^, 55 µM. (b). Rs_4_ and Rs_11_, 3.00 mM. (c) Rs_11_: [Ru(dmbpy)_3_]^2+^ (6 : 1), 1.5 mM (for Rs_11_). (d). Rs_4_: [Ru(dmbpy)_3_]^2+^ (6 : 1), 1.28 mM (for Rs_4_). (e) Aliphatic/fluorinated-hydrophilic copolymers (A/F_*xx*_-Copoly), 0.2 mM and 0.872 mM. All stock solutions are less than a month old and were stored in darkness. The ratio concentration of Ru-complex to Rs_11/4_ is fixed at 10 µM: 60 µM while the copolymers ratio varied: 10 mol% (7 µM, 0.7 mol eq. to 1 cage), 50 mol% (60 µM, 1 mol eq. to 1 cage) and 90 mol% (540 µM, 54 mol eq. to 1 cage). Free guest (Ru-complex alone) is fixed at 10 µM. The samples were measured after incubation period and conditions as previously specified.

### Fluorescence emission spectra normalization and encapsulation% calculations

The following equations were used to calculate the encapsulation efficiencies are adapted from a previous work.^34^ The method to determine the stability of the cages upon addition of the copolymers is based on fluorescence-based encapsulation, with the percentages relying on a normalization procedure assuming that the reference state corresponds to fully encapsulated guest under specific conditions. The maximum emission of the free guest (RFU_627_) was observed at peak centre of 627 nm whether the copolymers are present or not (Fig. S1–S3 and Table S2). Whereas for the encapsulated guest; the maximum emission (RFU_595/593_) was observed around peak centre of 595 nm in [Rs_4_]_6_ cage and around 593 nm in [Rs_11_]_6_ cage at equilibrium state where maximum guest uptake is assumed (Fig. S4–S9 and Table S1). The ratio *R*_*x*_ is calculated as follows:
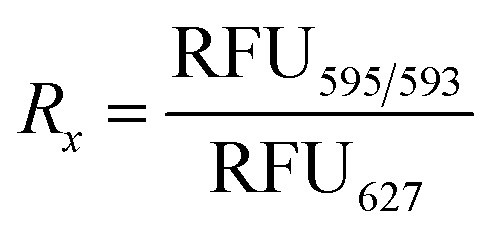
where *x* stands for: sample, free, encap; for the sample, free guest with copolymer sample and encapsulated guest in absence of copolymer sample, respectively. The percentage of encapsulated guest was normalized from 0 to 100%, denoting free guest and encapsulated guest in absence of copolymers, respectively. The encapsulation ratio *R*_encap_ is determined using the equilibrium state of a 1 : 1 ([Rs_4/11_]_6_ to guest) system. This calculation assumes maximum guest uptake occurs only when the hexamer cage remains structurally intact and free from external interference imposed by copolymer addition. The normalization and calculation of encapsulated guest% (*R*_encap_%) is as follows:
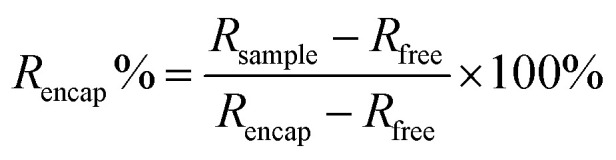


The calculated values are found in Table S1 and Table S2.

### Preparation of ^1^H- and DOSY-NMR cage–guest samples in deuterated organic solvents

Stock solutions of the cages [Rs_4_]_6_ and [Rs_11_]_6_ in deuterated organic solvents were made at concentrations ranging between 0.20–12.00 mM. Stock solutions of the alkyl ammonium bromide salts (tetrabutyl ammonium (TBA), tetrahexyl ammonium (THA) and tetraoctyl ammonium (TOA)) were made at different concentrations. The ratio of cage/salt was 1: 4–6. Samples were measured after >10 hours at 27 °C. To determine the ratio of cage-to-encapsulated guest in CDCl_3_, CD_2_Cl_2_, and toluene-d_8_, resonance shifts related to the cage (4.32–4.70 ppm, methine bridge, 24H) were integrated and set to 24 and compared with the integral value of a resonance shift belonging to the encapsulated guest (THA-Br) in the upfield region (−1.30 to −0.41 ppm, methyl, 12H). See Table S3 in SI. DOSY-NMR spectral data are found in Table S4.

### Preparation of DLS samples of cage–guest in organic solvents

Stock solutions of the cages [Rs_4_]_6_ and [Rs_11_]_6_ were made at concentrations ranging between 0.20–12.00 mM. Stock solutions of the alkyl ammonium bromide salts (tetrabutyl ammonium (TBA), tetrahexyl ammonium (THA) and tetraoctyl ammonium (TOA)) were made at different concentrations. The ratio of cage/salt was 1: 4–6. Samples were measured immediately after mixing with the ammonium salts and lasted between 10 hours up to 2 days. Taking 10 successive measurements after each hour. The detected size distributions are volume weighted. See Table S5.

## Results and discussion

We have synthesized resorcin[4]arene-based cages with two different lengths of footing side chains; butyl (C_4_H_9_ in *C*-butylcalix[4]resorcinarene, Rs_4_) and undecyl (C_11_H_23_ in *C*-undecylcalix[4]resorcinarene, Rs_11_) to tune their interactions with the surrounding media. The successful synthesis of the units (Rs_11_ and Rs_4_) has been proven through the detection of *m*/*z* intensities in ESI-ToF MS analysis (Fig. S43 and S44, respectively) showing the expected molecular weights of the macrocycles (Rs_11_ = 1105.68 g mol^−1^ and Rs_4_ = 712.92 g mol^−1^, respectively). ^1^H-NMR spectral analysis of both units in acetone-*d*_6_ show the assignments of the distinctive resonances of the macrocycles (Fig, S10). Previous studies have already established the formation of the hexamer cages ([Rs_4_]_6_ and [Rs_11_]_6_) in chloroform.^28^ Dynamic light scattering (DLS) analysis, summarized in Table S5, confirms that these cages display a hydrodynamic diameter (*D*_h_) between 2.0–3.9 nm, immediately upon dissolving in non-hydrogen-bond competing media like chloroform (Table S5, CHCl_3_, index 1). This size indicates formation of the hexameric structure, especially when compared with the significantly smaller dimensions of 0.8–1.3 nm observed in hydrogen-bond competing media like THF (Table S5, THF, index 1), where the cages likely undergo a disassembly process.

**Fig. 3:**
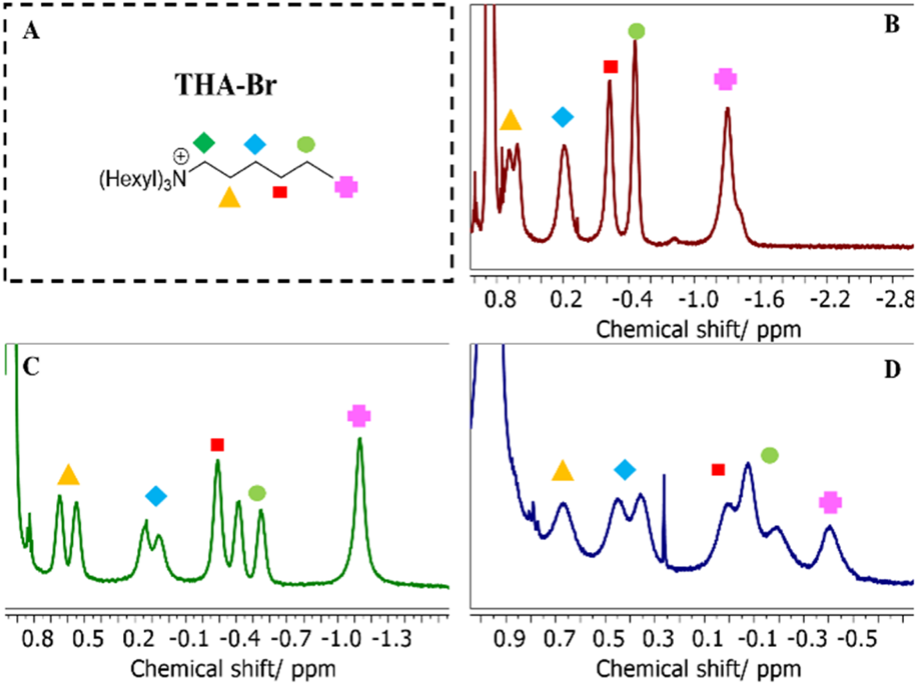
^1^H-NMR spectra of encapsulated THA-Br (A) in [Rs_11_]_6_ cage in CDCl_3_ (B), in DCM-d_2_ (C), and in toluene-d_8_ (D), showing the upfield shifts of the alkyl chains.

To systematically evaluate guest–cage stabilities, two guest models were chosen: an ammonium salt and a metal–ligand complex.^35^ The ammonium salt served as a probe for a successful confirmation of cage assembly by its confinement, and thus an indication of encapsulation into the [Rs_11_]_6_[Rs_4_]_6_ cages by the appearance of an characteristic upfield shift of resonances in ^1^H-NMR spectra in different organic media (Fig. 3B and D and S17, respectively), resulting from the shielding environment of the assembled hexamer cage interior.

Further a metal–ligand complex was employed to assess the cage's structural integrity when exposed to varying concentrations of random amphiphilic copolymers.

The hydrophobic segments of the copolymers may interact with the cage's aliphatic “footing” side chains, the hydrophilic component (PEG side chains) poses a risk of competitive interference with the hydrogen-bonding network that maintains the cage's architecture. By monitoring the specific fluorescence emission changes of the chromophoric metal–ligand guest, the efficiency of encapsulation in nonpolar media was used as the primary measure to determine how these competing interactions influence the overall stability of the assembly.

Copolymers can self-assemble into different assemblies, such as SCNPs or micelles, using water and other selective solvents.^39–46^ To explore the potential of incorporating hexamer cages into such polymer assemblies as isolated reaction sites or carriers, random copolymers were introduced to the cage–guest system in organic media. This serves as a pre-step to allow for a detailed investigation how different copolymer segments, including PEG, aliphatic, and fluorinated side chains, influence the structural integrity, stability, and encapsulation efficiency of the cages.

The copolymers are designed to balance the influence of their solvophilicity on the resulting nanostructures once present in aqueous media. Methacrylate monomers are used with pendant side chains of different affinities: poly(ethylene glycol) methyl ether methacrylate (PEGMA) monomers display hydrophilic PEG chains, whereas hydrophobic segments are formed by either *n*-butyl methacrylate (BuMA) in the aliphatic-hydrophilic copolymer (A*_x_*-Copoly), or by heptafluorobutyl methacrylate (HFBMA) in the fluorinated-hydrophilic copolymer (F*_x_*-Copoly). Systematic variations in the hydrophobic content generate copolymers with a hydrophobic content from 10 mol% (A/F_10_-Copoly) to 30 mol% (A/F_30_-Copoly). An incorporated azide-group-functionalized monomer is a potential crosslinking moiety for future applications beyond the scope of this investigation (Fig. 2).^36^

Given that the copolymers have 10–30 mol% hydrophobic content in their composition, it is assumed that their conjugation with resorcin[4]arene cages can improve their overall stability by interacting with the hydrophobic side chains of the cage. This potentially can broaden their applications in different surrounding media, knowing that amphiphilic copolymers can self-assemble into various nanostructures like single chain nanoparticles (SCNPs), crosslinked nanogels or micelles.^47–51^

### Encapsulation of alkylammonium guests in organic solvents

**Fig. 4:**
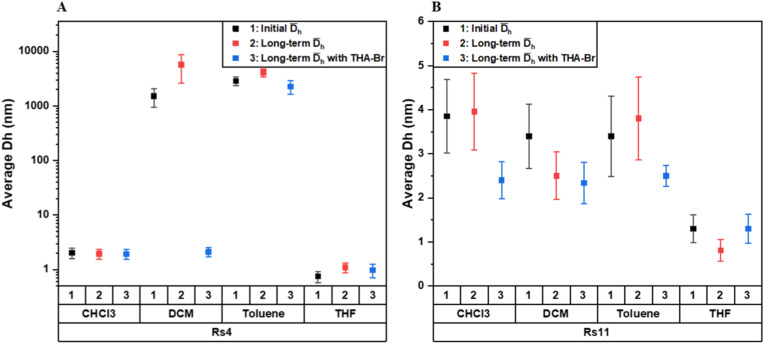
Collected DLS data of size change analysis of [Rs_4_]_6_ (A) and [Rs_11_]_6_ (B) in different organic solvents (chloroform: CHCl_3_, dichloromethane: DCM, toluene and tetrahydrofuran: THF). Data were collected immediately after dissolution (index 1) and over long periods of time (from 10 hours up to 2 days), in absence (index 2) and presence (index 3) of THA-Br. See Table S5.

We firstly investigated the encapsulation of alkylammonium guests by the [Rs_11_]_6_ cages with exchange of solvent molecules *via* NMR studies, based on methods reported by Rebek and coworkers.^19^ In chloroform, the cages ([Rs_4_]_6_ and [Rs_11_]_6_) predominantly assemble as hexameric species, with each unit adopting a crown configuration.^52^ The stability of both cages is high in the presence of encapsulated guest molecules. The measured hydrodynamic diameters (*D*_h_) of both cages are detailed in Table 1, determined by DLS size analysis and DOSY-NMR-analysis (before and after adding the guest molecule THA-Br, Fig. S20 and S24, respectively) using standard methods. They remain largely unchanged upon addition of alkylammonium salts in chloroform. Specifically, DOSY-NMR analysis for [Rs_11_]_6_ indicates a stable diameter of 3.6 nm, both before and after the introduction of tetrahexylammonium bromide (THA-Br) (Table 1, indices 1 and 2, DOSY and Fig. S20 and S24). While DLS size analysis recorded a slight reduction in size from 4.0 nm to 2.4 nm following the addition of the guest, they fall well within the expected dimensions for an intact hexamer cage, suggesting that the overall structural integrity is retained (Table 1, DLS and Fig. 4B, in CHCl_3_, entries 1&2).

**Table 1:** Collected data from DLS and DOSY-NMR spectra analysis for ([Rs_4_]_6_ and [Rs_11_]_6_ in organic solvents. ^*^The entries represent the hydrodynamic diameters in nm after equilibration; in the presence of THA-Br (✓) as a guest (G) and in its absence (✗). (I): index. (NA): not applicable. See Table S4 and S5 in SI

Solvents	[Rs_4_]_6_				[Rs_11_]_6_	
	I	G	^*^DLS/*D*_h_	^*^DOSY/*D*_h_	^*^DLS/*D*_h_	^*^DOSY/*D*_h_
CHCl_3_	1	✗	1.9	2.6	4.0	3.6
	2	✓	1.9	2.4	2.4	3.6
DCM	3	✗	5703	NA	2.5	NA
	4	✓	2.1	NA	2.3	NA
Toluene	5	✗	4094	NA	3.8	3.3
	6	✓	2251	NA	2.5	4.7

Similarly, [Rs_4_]_6_ showed a *D*_h_ of 2.6 nm with THA-Br and of 2.4 nm in its absence (Table 1, indices 1 and 2, DOSY and Fig. S20 and S24), while no change has been detected by DLS over long-term measurements (Table 1, indices 1 and 2, DLS and Fig. 4A, CHCl_3_, indices 2 and 3). This suggests that the intrinsic stability of both cages is mainly dictated by their own internal structure, stabilized by the hydrogen-bonding interactions with the embedded water molecules. These stable size distributions were also detected immediately after dissolution/mixing and persisted over long periods of time (see SI: Fig. S28–S31).

^1^H-NMR spectra in CDCl_3_ for [Rs_11_]_6_ (Fig. 3B) and [Rs_4_]_6_ (Fig. S17A) show upfield shifts belonging to encapsulated THA-Br, as a proof of successful cage formation and guest encapsulation.

In dichloromethane (DCM), a significant difference in stability emerges between the two cages, highlighting the critical role of the guest molecule for cage formation and its long-term stability. According to DLS size analysis, cage [Rs_11_]_6_ demonstrates a good stability in DCM with a *D*_h_ = 3.4 nm immediately upon dissolution (Table 1, indices 3 and 4, DLS Fig. 4B, in DCM). This assembly persists over time, averaging a *D*_h_ = 2.5 nm after 10 hours (Table 1, index 3, DLS and Fig. 4B, DCM, index 2). Furthermore, adding THA-Br to the cage retains a *D*_h_ of 2.3 nm (Table 1, index 4, DLS), indicative of stable hexamer assembly. This is further supported by fitted ^1^H-DOSY NMR spectra of the cage–guest complex, which display distinct diffusion peaks for the encapsulated THA-Br that align with the diffusion profile of the hexamer cage [Rs_11_]_6_ (Fig. S25).

In contrast, the hexameric cage, [Rs_4_]_6_ in DCM only displays structural stability upon addition of the alkylammonium salt, which yields a consistent *D*_h_ of 2.1 nm, that then remains unchanged over 2 days (Table 1, index 4, DLS and Fig. 4A, DCM, index 3). Without the presence of a guest molecule, Rs_4_ fails to form the desired hexamer; instead, it generates large, unstable assemblies with diameters ranging from 2500 to 9400 nm and over long periods of time (Table 1, index 3, DLS and Fig. 4A, indices 1 and 2, in DCM, and SI; Fig. S32). While the exact structure of these aggregates remains unclear, their size suggests they are the result of non-specific precipitation and uncontrolled clustering rather than a selective self-assembly process that yields a discrete [Rs_4_]_6_ hexamer cage.

The ^1^H-NMR spectrum of [Rs_11_]_6_ in DCM-d_2_ shows upfield shifts between −1.13–0.65 ppm of the encapsulated THA-Br (1 guest in an assumed staggered conformation per cage as reported in literature,^28^ see Table S3), confirming successful cage formation and guest encapsulation (Fig. 3C). The ^1^H-NMR spectrum of [Rs_4_]_6_ confirms the presence of the salt-templated [Rs_4_]_6_ hexamer cages *via* upfield shifts between (−1.16 – 0.6 ppm) of the encapsulated THA-Br (one guest per cage, in an assumed staggered conformation per cage as reported in literature,^28^ see Table S3), which proves successful cage formation and guest encapsulation (Fig. S17, B). This observation is further corroborated by the fitted ^1^H-DOSY NMR spectra of the [Rs_4_]_6_ cage–guest complex, which reveals distinct diffusion peaks for the encapsulated THA-Br. These peaks align with the diffusion profile of the hexamer [Rs_4_]_6_ confirming that the guest molecules are moving as a single unit together with the host framework (Fig. S25).

However, accurate determinations of *D*_h_ of both cages *via* DOSY-NMR were not possible in DCM-d_2_ due to a strong increase in solvent viscosity, evidenced by the diffusion coefficient of DCM-d_2_, which increased significantly from the expected value of 10^−5^ to 10^−8^ cm^2^ per sec. Such a profound change suggests that hexamer structures are no longer present, rather substituted by slow-moving larger aggregates (without THA-Br, Fig. S21 and with THA-Br, Fig. S25).

Also in toluene, the difference in stability is significant. According to size analysis by DLS, [Rs_11_]_6_ exhibits increased stability compared to [Rs_4_]_6_ under identical conditions. The [Rs_11_]_6_ hexameric cage forms immediately (*D*_h_ = 3.4 nm, Fig. 4B, toluene, index 1) and persists over long periods of time (*D*_h_ = 3.8 nm, Fig. 4B, toluene, index 2), indicative that [Rs_11_]_6_ can be stabilized in toluene independent of the presence of alkylammonium guests (Table 1, index 5, DLS). Fig. 3D displays the ^1^H-NMR spectrum of [Rs_11_]_6_ in toluene-d_8_ showing the characteristic upfield shifts of encapsulated THA-Br in the stable cage. This conclusion is further supported by the size analysis of ^1^H-DOSY NMR spectra of [Rs_11_]_6_ cage–guest complex. The reported dimensions ranging between 3.3–4.7 nm, are consistent with the expected size of a discrete hexamer structure in the solution state (Table 1, indices 5 and 6, DOSY and Fig. S26).

On the other hand, the [Rs_4_]_6_ hexamer could not be stabilized as dominant species in toluene upon dissolution, but rather formed larger aggregates (2251–4094 nm) that persisted over long periods of time as dominant species despite the presence of THA-Br (Table 1, indices 5 and 6, DLS and Fig. 4A, toluene, indices 1, 2 and 3). Although DLS size analysis suggests a lack of hexamer formation due to the presence of large aggregates, ^1^H-NMR spectrum analysis of [Rs_4_]_6_ cage–guest system in toluene-d_8_ provides more evidence of cage formation. The appearance of upfield resonance shifts for the encapsulated THA-Br confirms the successful formation of the [Rs_4_]_6_ cage (Fig. S17C). Although this indicates the presence of the cage, it likely represents a minor fraction of the sample, while the main parts of the material form larger aggregates due to further assembly in toluene.

Thus, the comparative analysis leads to the clear finding that [Rs_11_]_6_ possesses a high stability across all investigated solvents, capable of self-assembling and maintaining its hexamer structure. Furthermore, we find that the longer alkyl chain (undecyl) plays a major rule in this stabilizing effect. [Rs_4_]_6_ exhibits conditional stability that is highly dependent on the solvent and the presence of the guest molecule. While it shows high stability in chloroform, its assembly in DCM is templated by the alkylammonium salt. In toluene, [Rs_4_]_6_ fails to form the desired hexamer cage and instead forms large, non-hexamer aggregates independent of the presence of guests.

In tetrahydrofuran (THF), both Rs_11_ and Rs_4_ display a *D*_h_ between 0.8–1.3 nm (Fig. 4A and B, THF, indices 1, 2 and 3). However, ^1^H-NMR spectral analysis revealed the absence of upfield shifts for THA-Br, indicating that there is no encapsulated species (Fig. S19 and S27). This lack of assembly is attributed to the polar nature of THF, which disrupts the necessary hydrogen bonding required for hexamer formation.

### Compatibility and effects of amphiphilic random copolymers on cage–guest complexes in chloroform

The cages underwent further evaluation in view of their stability and functionality of encapsulating a chromophore guest. The choice as a guest model was a ruthenium complex; tris(4,4′-dimethyl-2,2′-bipyridine) ruthenium bis(hexafluorophosphate) (Ru(dmbpy)_3_(PF_6_)_2_) or [Ru(dmbpy)_3_]^2+^ (Fig. 3A) as a tool to monitor/quantify the cage's encapsulation efficiency.^35^ It is stabilized by cation–π interactions within the cage and templates the internal space due to its geometry, so facilitating cage formation and stability.^12,53^ The aliphatic-hydrophilic copolymers (A*_x_*-Copoly) and fluorinated-hydrophilic copolymers (F*_x_*-Copoly) (Fig. 2), were employed to gain deeper understanding of their impact on the cage–guest system. The large hydrophilic fraction (70–90 mol%) of the copolymers enhances water solubility and dispersibility; whereas the hydrophobic parts is projected to interact with the cages' hydrophobic alkyl-chains (butyl or undecyl, Fig. 1). Thus, the smaller hydrophobic fraction (10–30 mol%), will interact with the cage by associating with the cage's surface, so creating the necessary hydrophobic water-deficient compartments within polymeric single chains for future application.^7,34^

**Fig. 5:**
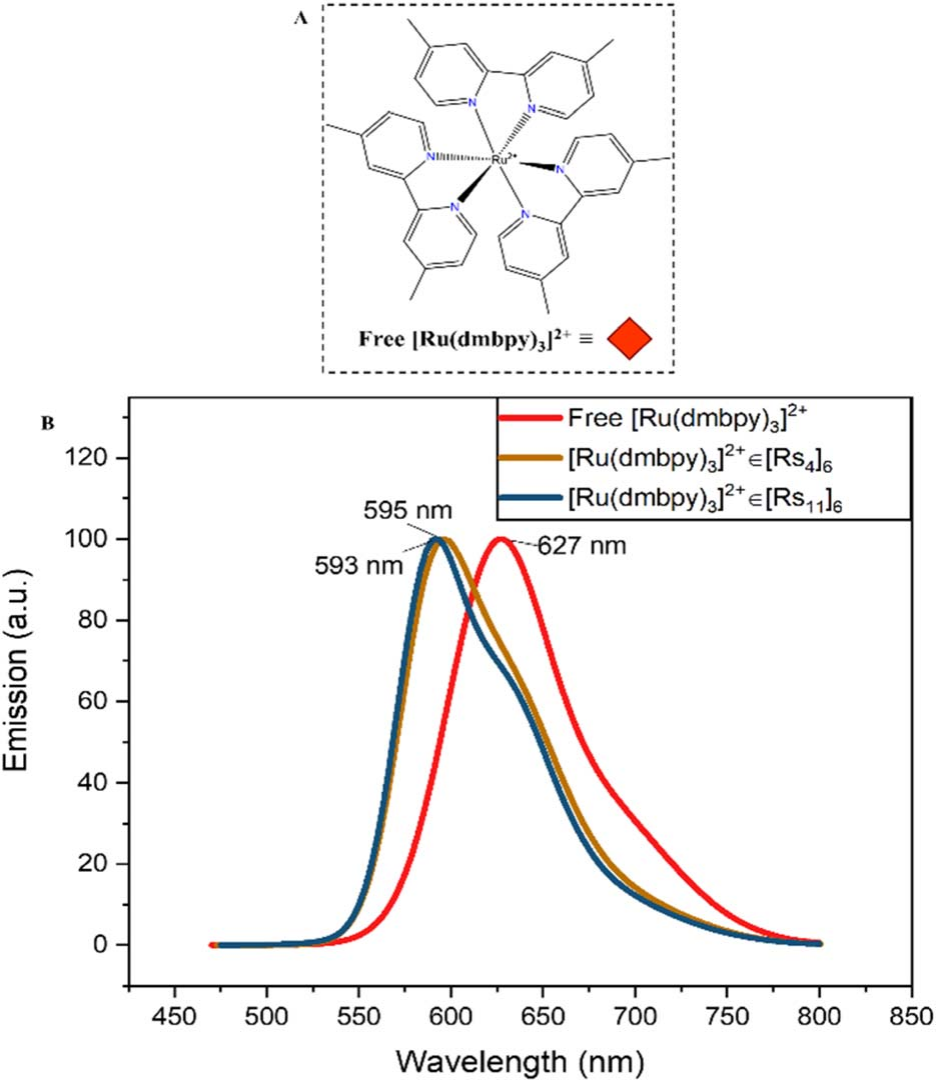
Fluorescence emission spectra of free [Ru(dmbpy)_3_]^2+^ complex, encapsulated [Ru(dmbpy)_3_]^2+^ in [Rs_4_]_6_ ([Ru(dmbpy)_3_]^2+^*ε*[Rs_4_]_6_) and encapsulated [Ru(dmbpy)_3_]^2+^ in [Rs_11_]_6_ ([Ru(dmbpy)_3_]^2+^*ε*[Rs_11_]_6_) in chloroform (B). The structure of [Ru(dmbpy)_3_]^2+^ (A) helps templating the cages.

**Fig. 6:**
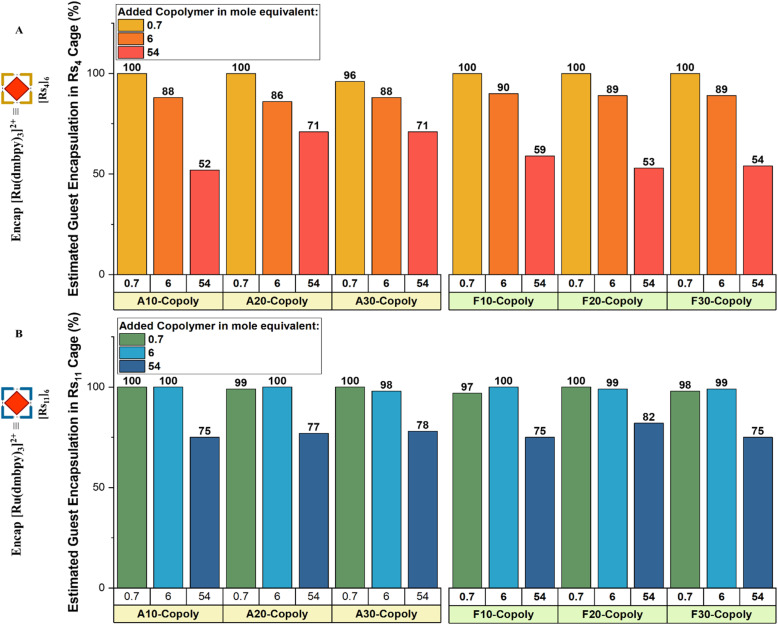
Estimated guest encapsulation in % of [Rs_4_]_6_ in presence of amphiphilic copolymers (A) and in [Rs_11_]_6_ in presence of amphiphilic copolymers (B) at different concentrations. The data was extracted from fluorescence emission spectra.

We used changes in the guest-specific absorption and emission spectra in organic media to monitor the stability of the cages and to prove the self-assembly of the functional cage in interaction with the copolymers.^54^ Encapsulation of [Ru(dmbpy)_3_]^2+^ within [Rs_4_]_6_ and [Rs_11_]_6_ results in a significant blue shift in its fluorescence emission, so allowing to determine the fraction of encaged/free complex. Free [Ru(dmbpy)_3_]^2+^ in absence of the cages in chloroform displays an absorption wavelength around *λ*_ab_ = 460 nm and a maximum fluorescence emission around *λ*_em_ = 627 nm (Fig. 5B). These values remain unchanged in the presence of copolymers alone (Fig. S1–S3), confirming that there is no significant interaction between the free Ru-complex and segments of the copolymers. However, upon encapsulation, the emission maximum shifts to *λ*_em_ = 595 nm in [Rs_4_]_6_ and *λ*_em_ = 593 nm in [Rs_11_]_6_, denoting the maximum guest uptake inside the cages under equilibrium conditions (Fig. 5B). Encapsulation and release capabilities of the cages in chloroform were evaluated using absorption and emission spectroscopy, performed on a fixed 1 : 1 cage–guest ratio across all measurements, comparing free and encapsulated guest spectra at equilibrium to probe the influence of the copolymer's composition with increasing hydrophobicity (aliphatic-hydrophilic copolymers: A_10_-Copoly, A_20_-Copoly and A_30_-Copoly and their fluorinated-counterparts: F_10_-Copoly, F_20_-Copoly and F_30_-Copoly, for their chemical structure see Fig. 2). The different copolymers were introduced at varying concentrations: 0.7 mol equivalent of copolymer, 6 mol equivalent of copolymer or 54 mol equivalent of copolymer *vs.* 1 cage. Fig. 6 shows the fraction of the encapsulated guest% (*R*_encap_%) with respect to 100% as maximum guest uptake at equilibrium.

At low copolymer content (0.7 mol eq.); both cages maintained high structural integrity, with encapsulation efficiencies exceeding 96%. At this concentration, it is concluded that the copolymers do not significantly interfere with the cage structure (Fig. 6A and B, 0.7 mol). At moderate copolymer content (6.0 mol eq.), a difference in stability became apparent. While [Rs_11_]_6_ remained stable with a 98–100% encapsulation efficiency, [Rs_4_]_6_ showed a slight decline to 86–90% encapsulation efficiency (Fig. 6A and B, 6.0 mol), indicative of an at least partially increased dynamics of this cage due to the interaction with the added copolymer(s).

At high copolymer content (54 mol eq.), a significant destabilization occurred, revealing distinct differences between the two cages. With [Rs_4_]_6_, the aliphatic copolymers (20–30 mol% hydrophobic content) preserved stability better (up to 71% encapsulation efficiency) in comparison to their fluorinated counterparts (up to 54% encapsulation efficiency) (Figure 6A, 54.0 mol). On the other hand, the [Rs_11_]_6_ cage exhibited superior overall stability (75–82% encapsulation), with no significant difference observed between aliphatic and fluorinated affinities (Figure 6B, 54.0 mol).

It is obvious that the [Rs_11_]_6_ cage is significantly more robust, maintaining its structural integrity to a higher amount of added copolymer. These findings correlate with previous analyses in different solvents, indicating that the [Rs_11_]_6_ cages are inherently more robust than the [Rs_4_]_6_ cages. The observed destabilization at higher concentrations (54 mol eq. of added copolymers) likely stems from PEG side chains competing for hydrogen-bonding sites with the water molecules incorporated within the cage structure (Fig. 2).

## Conclusion

The comparative analysis by DLS and DOSY-NMR spectra shows a distinct stability of two cages, bearing different alkyl-chains across different media. Thus, the cage [Rs_11_]_6_ displays a higher stability across all tested solvents in comparison to [Rs_4_]_6_, maintaining its hexamer structure even in the absence of the templating guests in solvents like toluene. In contrast [Rs_4_]_6_ shows a limited stability that is highly dependent on the solvent and the presence of the guest molecule, with both cages being unstable in THF. Emission spectroscopy in organic media revealed distinct stability profiles for the cages upon addition of amphiphilic copolymers featuring varying aliphatic and fluorinated co-monomer contents (10–30 mol%). Although both cages preserve their structural integrity effectively at low concentrations of the added copolymers, their structural integrity changes significantly at higher copolymer concentrations. The [Rs_4_]_6_ cage is more sensitive to the chemical nature of the copolymer, displaying a reduced structural integrity with fluorinated copolymers than towards the aliphatic copolymers, as evidenced by increased guest release. In contrast, [Rs_11_]_6_ shows a higher stability, remaining largely unaffected by the specific chemical composition of the added copolymers. The decrease in encapsulation efficiency at higher amounts of the added copolymer is attributed to a competitive mechanism, where the PEG side chains of the copolymers compete for the hydrogen-bonding sites typically occupied by the structural water molecules that stabilize the cage framework. Ultimately, the data confirms that [Rs_11_]_6_ is the more resilient cage. Its inherent robustness and ability to resist competitive H-bonding by the added PEG chains makes it an ideal candidate for the development of more complex, hierarchical nano-assemblies involving copolymers and cages in different solvents, even under high-load conditions.

## Author contributions

Wolfgang H. Binder has contributed in project planning & conceptualization, defining and adapting research goals, in addition to his supervision in inducing progress. He also contributed in reviewing and editing the content of this publication. Marah Alqaisi has contributed in developing and modifying quantitative and qualitative experimental methodologies, analysing and assessing data to uncover and identify patterns between variables, integrating and aggregating data, reporting findings and progress. In addition, monitoring and reporting progress, researching and reviewing literature, drafting and creating the full version of this article with its supplementary materials and its visual aids to its final form.

## Conflicts of interest

There are no conflicts to declare.

## Supplementary Material

RA-016-D6RA01475E-s001

## Data Availability

All original data are stored in our electronic lab notebook (E-lab), where the original data can be retrieved on demand. Organization of these data inside the system is according to the rules of the DFG (German Research Foundation). This access can be granted on request, but cannot be provided by an open link, to secure other data inside the system. We will provide all data on 1H-NMR, 13C-NMR, Tensile Testing, CP-MAS, TGA, FT-IR, TEM and BDS electronically on demand. Supplementary information (SI) is available. See DOI: https://doi.org/10.1039/d6ra01475e.

## References

[cit1] Insua I., Montenegro J. (2020). Chem.

[cit2] Koblenz T. S., Wassenaar J., Reek J. N. H. (2008). Chem. Soc. Rev..

[cit3] Ljubetič A., Lapenta F., Gradišar H., Drobnak I., Aupič J., Strmšek Ž., Lainšček D., Hafner-Bratkovič I., Majerle A., Krivec N., Benčina M., Pisanski T., Veličković T. Ć., Round A., Carazo J. M., Melero R., Jerala R. (2017). Nat. Biotechnol..

[cit4] Maiti S., Fortunati I., Ferrante C., Scrimin P., Prins L. J. (2016). Nat. Chem..

[cit5] Urban P., Pritzl S. D., Konrad D. B., Frank J. A., Pernpeintner C., Roeske C. R., Trauner D., Lohmüller T. (2018). Langmuir.

[cit6] Izgu E. C., Björkbom A., Kamat N. P., Lelyveld V. S., Zhang W., Jia T. Z., Szostak J. W. (2016). J. Am. Chem. Soc..

[cit7] Zappacosta R., Aschi M., Ammazzalorso A., Di Profio P., Fontana A., Siani G. (2019). Biochim. Biophys. Acta Biomembr..

[cit8] Feher K. M., Hoang H., Schramm M. P. (2012). New J. Chem..

[cit9] Rhlalou T., Ferhat M., Frouji M. A., Langevin D., Métayer M., Verchère J. F. (2000). J. Membr. Sci..

[cit10] MacGillivray L. R., Atwood J. L. (1997). Nature.

[cit11] Borsato G. and Scarso A., in Organic Nanoreactors, ed. S. Sadjadi, Academic Press, Boston, 2016, 10.1016/b978-0-12-801713-5.00007-0, pp. 203–234

[cit12] Beyeh N. K., Kogej M., Åhman A., Rissanen K., Schalley C. A. (2006). Angew. Chem., Int. Ed..

[cit13] Niederl J. B., Vogel H. J. (1940). J. Am. Chem. Soc..

[cit14] Zhang Q., Tiefenbacher K. (2013). J. Am. Chem. Soc..

[cit15] Zhang Q., Tiefenbacher K. (2015). Nat. Chem..

[cit16] Zhang Q., Catti L., Pleiss J., Tiefenbacher K. (2017). J. Am. Chem. Soc..

[cit17] Zhang Q., Rinkel J., Goldfuss B., Dickschat J. S., Tiefenbacher K. (2018). Nat. Catal..

[cit18] Gaeta C., Talotta C., De Rosa M., La Manna P., Soriente A., Neri P. (2019). Chem.--Eur. J..

[cit19] Shivanyuk A., Rebek J. (2001). Proc. Natl. Acad. Sci. U. S. A..

[cit20] Young M. C., Djernes K. E., Payton J. L., Liu D., Hooley R. J. (2019). J. Chem. Educ..

[cit21] Bianchini G., Sorella G. L., Canever N., Scarso A., Strukul G. (2013). Chem. Commun..

[cit22] La Sorella G., Sperni L., Strukul G., Scarso A. (2015). ChemCatChem.

[cit23] Catti L., Tiefenbacher K. (2015). Chem. Commun..

[cit24] Caneva T., Sperni L., Strukul G., Scarso A. (2016). RSC Adv..

[cit25] Cavarzan A., Scarso A., Sgarbossa P., Strukul G., Reek J. N. H. (2011). J. Am. Chem. Soc..

[cit26] Barrett E. S., Dale T. J., Rebek J. (2007). J. Am. Chem. Soc..

[cit27] Barrett E. S., Dale T. J., Rebek J. (2008). J. Am. Chem. Soc..

[cit28] Yamanaka M., Shivanyuk A., Rebek J. (2004). J. Am. Chem. Soc..

[cit29] Shivanyuk A., Rebek J. (2003). J. Am. Chem. Soc..

[cit30] Evan-Salem T., Baruch I., Avram L., Cohen Y., Palmer L. C., Rebek J. (2006). Proc. Natl. Acad. Sci. U. S. A..

[cit31] Hkiri S., Steinmetz M., Schurhammer R., Sémeril D. (2022). Chem.--Eur. J..

[cit32] Ichou H., Telliez K., Lajnef S., Peyrot F., Doistau B., Leherte L., Colasson B. (2025). Inorg. Chem. Front..

[cit33] Tanaka Y., Kobuke Y., Sokabe M. (1995). Angew Chem. Int. Ed. Engl..

[cit34] Muthwill M. S., Bina M., Paracini N., Coats J. P., Merget S., Yorulmaz Avsar S., Messmer D., Tiefenbacher K., Palivan C. G. (2024). ACS Appl. Mater. Interfaces.

[cit35] Matiz C., Castellanos K., Maldonado M. (2025). Processes.

[cit36] Alqaisi M., Thümmler J. F., Lehmann F., Schmitt F. J., Lentz L., Rieder F., Hinderberger D., Binder W. H. (2024). Polym. Chem..

[cit37] Chwastek M., Cmoch P., Szumna A. (2022). J. Am. Chem. Soc..

[cit38] Elidrisi I., Negin S., Bhatt P. V., Govender T., Kruger H. G., Gokel G. W., Maguire G. E. M. (2011). Org. Biomol. Chem..

[cit39] Hoffmann J. F., Roos A. H., Schmitt F. J., Hinderberger D., Binder W. H. (2021). Angew Chem. Int. Ed. Engl..

[cit40] Thummler J. F., Maragani R., Schmitt F. J., Tang G., Rahmanlou S. M., Laufer J., Lucas H., Mader K., Binder W. H. (2023). Chem. Commun..

[cit41] Thummler J. F., Roos A. H., Kruger J., Hinderberger D., Schmitt F. J., Tang G., Golmohamadi F. G., Laufer J., Binder W. H. (2023). Macromol. Rapid Commun..

[cit42] Thümmler J. F., Binder W. H. (2024). Chem. Commun..

[cit43] Li Y., Deng Y., Tong X., Wang X. (2006). Macromolecules.

[cit44] Dan K., Bose N., Ghosh S. (2011). Chem. Commun..

[cit45] Zhu X., Liu M. (2011). Langmuir.

[cit46] Forster S., Plantenberg T. (2002). Angew Chem. Int. Ed. Engl..

[cit47] Kedar U., Phutane P., Shidhaye S., Kadam V. (2010). Nanomed. Nanotechnol. Biol. Med..

[cit48] Kröger A. P. P., Hamelmann N. M., Juan A., Lindhoud S., Paulusse J. M. J. (2018). ACS Appl. Mater. Interfaces.

[cit49] Hamelmann N. M., Paats J.-W. D., Paulusse J. M. J. (2021). ACS Macro Lett..

[cit50] Sanchez-Sanchez A., Akbari S., Etxeberria A., Arbe A., Gasser U., Moreno A. J., Colmenero J., Pomposo J. A. (2013). ACS Macro Lett..

[cit51] Delledonne A., Guazzelli E., Pescina S., Bianchera A., Galli G., Martinelli E., Sissa C. (2023). Acs Applied Nano Materials.

[cit52] Poole, III D. A., Mathew S., Reek J. N. H. (2021). J. Am. Chem. Soc..

[cit53] Bianchini G., Scarso A., Sorella G. L., Strukul G. (2012). Chem. Commun..

[cit54] Horiuchi S., Tanaka H., Sakuda E., Arikawa Y., Umakoshi K. (2016). Chem.--Eur. J..

