# Supplementary material for: Stability and compatibility of resorcin[4]arene hexamer cages with amphiphilic random copolymers in organic solvents
Source: RSC Adv. 2026 May 11;16(26):23662–72. doi: 10.1039/d6ra01475e (PMC13159794; doi:10.1039/d6ra01475e)
Supplement: RA-016-D6RA01475E-s001 [file RA-016-D6RA01475E-s001.pdf]

Supplementary Information (SI) for RSC Advances.
This journal is © The Royal Society of Chemistry 2026

Supporting Information

Exploring Resorcin[4]arene Hexamer Cages: Stability and Compatibility with Amphiphilic Random Copolymers in Organic Solvents

Marah Alqaisi [a] and Wolfgang H. Binder* [a]

[a] *Macromolecular Chemistry, Institute of Chemistry, Faculty of Natural Science II (Chemistry, Physics and Mathematics), Martin-Luther-University Halle-Wittenberg, von-Danckelmann-Platz 4, Halle D-06120, Germany.*

I. Experimental

i. Materials

Resorcinol (99%, Roth), dodecanal (95%, BLDpharm), pentanal (98.5%, Sigma-Aldrich), hydrochloric acid (37 w%, Grüssing), tetrahexylammonium bromide (THA-Br) (99%, Sigma-Aldrich), tetraoctylammonium bromide (TOA-Br) (Fluka), tetrabutylammonium bromide (TBA-Br) (>99%, TCI acetone-d_6 (99.8%, Chemotrade), tris(4,4'-dimethyl-2,2'-bipyridine)ruthenium bis(hexafluorophosphate) ($Ru(dmbpy)_3(PF_6)_2$) (95%, Sigma-Aldrich) , chloroform-d_3 ($CDCl_3$) (stabilized with Ag, ≤100%, ARMAR), toluene-d_8 (99.8%, ARMAR), dichloromethane-d_2 (95.5%, ARMAR), ethanol (99.8%, Fisher Chemical). Redistilled solvents: chloroform ($CHCl_3$), dichloromethane (DCM), tetrahydrofuran (THF), toluene, methanol. De-ionized water (from TKA-GenPure system).

ii. Methods

^1H- and ^{13}C- NMR spectra were recorded by using Agilent Technologies 400MHz VNMRS and Agilent Technologies 600 MHz shielded VNMRS spectrometers at 27°C in $CDCl_3$, toluene-d_8, DCM-d_2 and acetone-d_6. MestReNova (14.2.1-27684) was used as a software to analyse the spectra. ^1H-DOSY NMR was recorded by using Agilent Technologies 400MHz VNMRS spectrometer at 27°C the previously mentioned deuterated solvents with 1.0 ms – 3.0 ms as gradient time and 100 ms – 300 ms as diffusion time. MestReNova (14.2.1-27684) was used as a software to analyse the spectra. Dynamic light scattering (DLS) measurements were done using Litesizer 500 from Anton Paar with 10 mm low volume quarts cuvette. The irradiation wavelength was 658 nm with detection angles at 90° (side scattering) or at 175° (back scattering) or at 15° (forward scattering). The temperature was set to 25°C with varying sample concentrations in organic solvents. ESI-TOF-MS measurements were done with Bruker Daltonics microTOF via direct injection at a flow rate of 180 µL/hr in positive and negative modes with an acceleration voltage of 4.5 kV. Samples were prepared by dissolving in methanol or in acetone or in chloroform or in acetone/chloroform (1:2) (0.1 - 0.3 mg/mL), depending on the preparation methods as mentioned in subsequent sections. The instrument was calibrated using the ESI-L low concentration tuning mix from Agilent Technologies (product no. G1969-85000). The software Data Analysis (version 4.0) was used for evaluation. UV/VIS-absorption measurements were done with Perkin Elmer LAMBDA 365 UV/Vis Spectrophotometer using Helma analytics quartz glass cuvettes with diameter 10 mm and screw cap. Ru-complex ($Ru(dmbpy)_3(PF_6)_2$), the guest concentration was always 10 µM, resorcin[4]arenes at 60 µM (Ru-complex/resorcin[4]arenes: 1:6) with varying copolymers concentrations (resorcin[4]arenes/copolymers: 9:1 or 1:1). The cage–guest system was incubated overnight at 45°C and sonicated ~30 minutes before sample preparation. The samples were incubated overnight at 45°C before measurements. Samples size: 3 ml. The temperature was controlled and set at 25°C with

waiting time of 3-5 minutes before measurements. Emission spectra were measured on a Cary Eclipse fluorescence spectrometer of Agilent for the same samples used for UV/Vis spectroscopy with the same conditions. The acquired data was processed using OriginPro 2029 software. 3D structures of resorcin[4]arene unit and cage were drawn using UCSF ChimeraX version 1.10rc202505100155 (2025-05-10). https://www.rbvi.ucsf.edu/chimerax . The atomic distances were adapted from the reference.[1]

iii. Synthesis

1. Synthesis of C-Undecylcalix[4]resorcinarene (**Rs**$_{11}$)

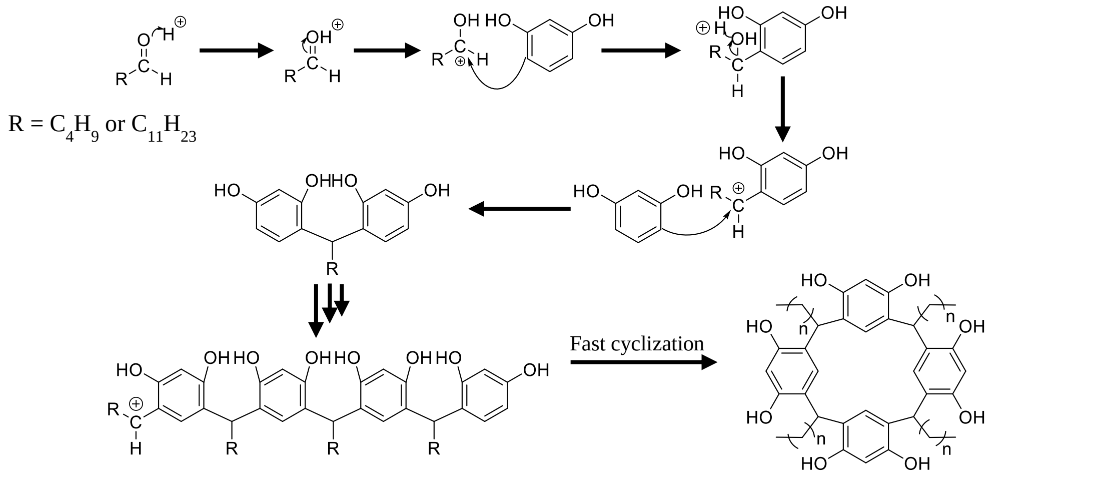

Hydrochloric acid (37w%, 13 ml, 90 mmol) was added to 38.3 mL of ethanol (99.8%) and stirred for few minutes. Resorcinol (10.07 g, 90 mmol) was added and the solution was brought to -1.7 to -1.5°C. Dodecyl aldehyde (20 ml, 90 mmol) was dissolved in 25 mL of ethanol (99.8%) and added drop-wise over 90 minutes using dropping funnel. The temperature was kept below 0°C and the solution was stirred through the addition process. After the addition of the aldehyde, the solution was brought to room temperature and placed in an oil bath. The temperature of the oil bath was increased until 100°C. The reaction was under reflux for 18 hours. The reaction was stopped by cooling down to room temperature and the product started precipitating as a yellow powder. The crude product was dispersed in cold methanol (-10°C) and filtered. The procedure was repeated 3 times until the supernatant was clear (total 400 mL). The yellowish powder was crystallized in methanol (54°C) and let to cool down to room temperature. It was then filtered with a solution of cold methanol/water (1:1) (300 mL) and then cold methanol (300 mL). The slight yellowish powder was dried at 45°C at 90 mbar for 2 hours. The product was collected in a container that is open to atmospheric air to let it stay moisturized. The product was confirmed using ESI-ToF and ^{1}H-NMR in acetone-d$_6$. Yield = 60%. The method was adopted and modified from the references.[2, 3] M = 1105.68 g/mol.

^1H-NMR (402 MHz, acetone-d_6, δ in ppm): 8.41 (s, 6H), 7.52 (s, 3H), 6.22 (s, 4H), 4.29 (s, 4H), 2.77 (s, 3H), 2.29 (s, 8H), 1.29 (s, 75H), 0.88 (s, 12H).

^{13}C-NMR (101 MHz, acetone-d_6, δ in ppm): 205.17, 151.79, 124.48, 124.29, 102.78, 33.48, 33.42, 31.77, 29.69, 29.60, 29.58, 29.54, 29.47, 29.34, 29.28, 29.23, 29.14, 29.09, 28.95, 28.89, 28.76, 28.71, 22.44, 13.46.

2. Synthesis of C-butylcalix[4]resorcinarene (**Rs_4**)

Hydrochloric acid (37w%, 0.83 ml, 10 mmol) was added to 20 mL of ethanol (99.8%) and stirred for few minutes in a cold-water bath (≈ 7°C). Resorcinol (1.1 g, 10 mmol) was added and the solution was brought to -1.0°C. Pentanal (1.06 mL, 10 mmol) was dissolved in 12 mL of ethanol (99.8%) and added drop-wise over 45 minutes using dropping funnel. The temperature was kept below 0°C and the solution was stirred through the addition process. After the addition of pentanal solution, the solution was brought to room temperature and placed in an oil bath. The temperature of the oil bath was increased until 100°C. The reaction was under reflux for 18 hours. The reaction was stopped by cooling down to room temperature and the amount of ethanol was reduced by evaporation. The highly-concentrated solution was precipitated in water twice (total 600 mL) and filtered until the supernatant was neutral. The slightly-yellowish powder was crystallized in methanol and washed with water (300 mL). The slight yellowish powder was dried at 40°C at 40 mbar for 45 minutes. The product was collected in a container that is open to atmospheric air to let it stay moisturized. The product was confirmed using ESI-ToF and ^1H-NMR in acetone-d_6. Yield was not calculated because product was wet. The method was adopted and modified from the references.[2, 3] M = 712.92 g/mol.

^1H-NMR (402 MHz, acetone-d_6, δ in ppm): 8.41 (s, 7H), 7.56 (s, 4H), 6.24 (s, 4H), 4.30 (s, 4H), 2.29 (s, 8H), 1.38 (s, 8H), 1.28 (s, 9H), 0.89 (s, 13H).

^{13}C-NMR (101 MHz, acetone-d_6, δ in ppm): 206.12, 152.72, 125.49, 125.29, 103.72, 34.33, 34.06, 31.31, 29.84, 23.37, 14.56.

iv. Preparation of Cage–guest System for Absorption and Emission Spectroscopy

Stock solution in $CHCl_3$ were prepared with the following concentrations:

1. $[Ru(dmbpy)_3]^{2+}[(PF_6)_2]^{2-}$, 55 µM (0.99 mg, 0.13 µmol)
2. Rs_4, 3.00 mM (36.76 mg, 51.56 µmol)
3. Rs_{11}, 3.00 mM (57.0 mg, 51.56 µmol)
4. Rs_4: $[Ru(dmbpy)_3]^{2+}$ (6:1), 1.493 mM in total (25.8 µmol: 4.3 µmol, respectively, in 20.2 ml).
5. Rs_{11}: $[Ru(dmbpy)_3]^{2+}$ (6:1), 1.5 mM in total (30.39 µmol: 5.07 µmol, respectively, in 20.3 ml).
6. Aliphatic/fluorinated-hydrophilic copolymers (**A_x-Copoly** and **F_x-Copoly**, respectively), 0.2 mM or 0.872 mM.

All of the stock solutions and samples were kept in incubation overnight (>15 hours) between 45°C with stirring and sonication before sample preparations. They were stored in darkness.

The ratio concentration of Ru-complex to $Rs_{11/4}$ is fixed at 10 µM: 60 µM while the copolymers ratio varied:

1. 10 mol% (7 µM, 0.7 mol eq. to 1 cage).
2. 50 mol% (60 µM, 1 mol eq. to 1 cage).
3. 90 mol% (540 µM ,54 mol eq. to 1 cage).
4. Free guest (Ru-complex alone) is fixed at 10 µM.

Excitation wavelength was between 460 – 465 nm at 600 V.

Range of emission detection was set between 400 – 800 nm. The acquired data was processed using OriginPro 2029, 9.6.0.172 (Academic) software.

II. Supporting Tables and Figures

i. Table S1: Fluorescence Emission in Presence of Cages $[Rs_4]_6$ and $[Rs_{11}]_6$

Table S 1: Collected data from fluorescence emission spectroscopy of ***$[Ru(dmbpy)_3]^{2+}$*** *to estimate guest encapsulation in % of* ***$[Rs_4]_6$*** *and in* ***$[Rs_{11}]_6$*** *in presence of amphiphilic copolymers at 3 different concentrations.*

0.7 mol equivalent										
Copolymers	RFU_{627}		$RFU_{595/593}$		R_{free}		R_{sample}		Encap%	
	$[Rs_4]_6$	$[Rs_{11}]_6$	$[Rs_4]_6$	$[Rs_{11}]_6$	$[Rs_4]_6$	$[Rs_{11}]_6$	$[Rs_4]_6$	$[Rs_{11}]_6$	$[Rs_4]_6$	$[Rs_{11}]_6$
A_{10}-Copoly	103	231	138	342	0.57	0.52	1.34	1.48	100	100
A_{20}-Copoly	102	137	135	201	0.54	0.51	1.32	1.47	100	99
A_{30}-Copoly	112	139	154	206	0.54	0.51	1.29	1.48	96	100
F_{10}-Copoly	106	135	140	196	0.56	0.51	1.32	1.45	100	97
F_{20}-Copoly	104	137	137	203	0.59	0.54	1.32	1.48	100	100
F_{30}-Copoly	109	134	144	196	0.56	0.51	1.32	1.46	100	98
$[Ru(dmbpy)_3]^{2+}$ ϵ $[Rs]_6$	107	132	141	196	-	-	1.32	1.48	100	100
6 mol equivalent										
A_{10}-Copoly	98	150	122	222	0.53	0.48	1.24	1.48	88	100
A_{20}-Copoly	99	143	123	211	0.55	0.51	1.23	1.48	86	100

A_{30}-Copoly	95	139	118	203	0.51	0.47	1.24	1.46	88	98
F_{10}-Copoly	99	144	125	214	0.52	0.47	1.26	1.49	90	100
F_{20}-Copoly	106	144	133	211	0.55	0.49	1.25	1.47	89	99
F_{30}-Copoly	102	144	127	212	0.50	0.50	1.25	1.47	89	99
$[Ru(dmbpy)_3]^{2+}$ ϵ $[Rs]_6$	109	133.1	146	196.6	-	-	1.34	1.48	100	100
54 mol equivalent										
A_{10}-Copoly	65	189	61	231	0.50	0.46	0.94	1.22	52.4	74.5
A_{20}-Copoly	131	195	144	243	0.51	0.47	1.1	1.25	71.1	77.2
A_{30}-Copoly	140	200	149	250	0.51	0.48	1.1	1.25	71.1	77.5
F_{10}-Copoly	121	166	121	203	0.52	0.46	1.0	1.22	58.5	74.5
F_{20}-Copoly	71	133	67	173	0.95	0.46	0.95	1.3	53	82.4
F_{30}-Copoly	77	105	75	129	0.53	0.48	0.97	1.23	54.3	75
$[Ru(dmbpy)_3]^{2+}$ ϵ $[Rs]_6$	109.1	133	145.7	197	-	-	1.34	1.48	100	100

ii. Table S2: Fluorescence Emission in Absence of Cages $[Rs_4]_6$ and $[Rs_{11}]_6$

Table S 2: Collected data from fluorescence emission spectroscopy of ***$[Ru(dmbpy)_3]^{2+}$*** *to estimate* ***R_{free}***

0.7 mol equivalent					
Copolymers	RFU_{627}	$RFU_{595/593}$		R_{free}	
		$[Rs_4]_6$	$[Rs_{11}]_6$	$[Rs_4]_6$	$[Rs_{11}]_6$
A_{10}-Copoly	37	21	20	0.57	0.54
A_{20}-Copoly	41	22	21	0.54	0.51
A_{30}-Copoly	39	21	20	0.54	0.51
F_{10}-Copoly	41	23	21	0.56	0.51
F_{20}-Copoly	39	23	21	0.59	0.54
F_{30}-Copoly	39	22	20	0.56	0.51
$[Ru(dmbpy)_3]^{2+}$	37	20	14	0.54	0.48
6 mol equivalent					
A_{10}-Copoly	36	19.2	17.4	0.53	0.48

A_{20}-Copoly	35.6	19.6	18.2	0.55	0.51
A_{30}-Copoly	35.9	18.4	16.7	0.51	0.48
F_{10}-Copoly	34.2	17.9	16.1	0.52	0.47
F_{20}-Copoly	35.3	19.4	17.3	0.55	0.49
F_{30}-Copoly	35.3	19	17.8	0.54	0.50
$[Ru(dmbpy)_3]^{2+}$	31	15	17	0.54	0.48
54 mol equivalent					
A_{10}-Copoly	39.6	20	18.1	0.50	0.46
A_{20}-Copoly	42.9	22.1	20.3	0.51	0.47
A_{30}-Copoly	40.4	20.5	18.7	0.51	0.48
F_{10}-Copoly	37.4	19.3	17.3	0.52	0.46
F_{20}-Copoly	38.2	19.6	17.6	0.51	0.46
F_{30}-Copoly	41	21.8	19.8	0.53	0.48
$[Ru(dmbpy)_3]^{2+}$	37	20	14	0.54	0.48

iii. Table S3 and Table S4: ^{1}H- and DOSY-NMR spectral data of Cage–Guest Samples in Deuterated Organic Solvents

Table S 3: Chemical shifts and integral values of characteristic resonance peaks of the cages and encapsulated THA-Br in different organic solvents after analyzing ^{1}H-NMR spectra.

Solvent	**Rs_4**		**Rs_{11}**		Encapsulated THA in **Rs_4**		Encapsulated THA in **Rs_{11}**		ratio	
	Chemical shift[a]	Integral[a]	Chemical shift[a]	Integral[a]	Chemical shift[b]	Integral[b]	Chemical shift[b]	Integral[b]	$[Rs_4]_6$:THA	$[Rs_{11}]_6$:THA
$CDCl_3$	4.32	24.0	4.31	24.0	-1.36	9.96	-1.30	10.57	1:0.83	1:0.88
CD_2Cl_2	4.34	24.0	4.40	24.0	-1.17	10.4	-1.13	10.48	1:0.87	1:0.87
Toluene-d_8	4.65	24.00	4.68	24.0	-0.37	16.06	-0.41	5.46	1:1.3	1:0.50

a: chemical shift (in ppm) as reference and integral of methine proton of $[Rs_{4/11}]_6$. b: chemical shift (in ppm) and integral of methyl protons of encapsulated THA-Br.

*Table S 4: Average diffusion coefficients of **Rs_4** and **Rs_{11}** with and without THA-Br in different deuterated solvents.*

Solvent	**Rs_4**		**Rs_{11}**		$^2\eta$
	^1D, Alone	^1D, with THA-Br	^1D, Alone	^1D, with THA-Br	
$CHCl_3$	3.0 ± 0.05	3.3 ± 0.12	2.2 ± 0.02	2.2 ± 0.12	5.57
DCM	NA	81.9 ± 5.0	126 ± 8.2	7.01 ± 0.38	3.42

Toluene	NA	NA	3.03 ± 0.22	2.12 ± 0.11	4.38
THF	4.74 ± 0.86	4.2 ± 0.71	4.5 ± 0.16	4.2 ± 0.13	4.82

1: Average diffusion coefficient ***D*** (in x10^{-6} cm^2/s) as detected from the fitted DOSY-NMR spectra. 2: dynamic viscosity (**η**) of deuterated solvent in x10^{-4} Pa.s. NA: Not applicable. ***D*** were substituted in Stokes-Einstein equation ($\boldsymbol{D} = \frac{k_B T}{3\pi\eta D_h}$) to calculate $\boldsymbol{D_h}$ in Table 1.

iv. Table S5: DLS Data

Table S 5: Collected DLS data of size change analysis of $[Rs_4]_6$ and $[Rs_{11}]_6$ in different organic solvents. Data was collected immediately after dissolution over long periods of time (from 10 hours up to 2 days), in presence and absence of THA-Br

		Rs_4		Rs_{11}	
Solvent	Index	$\bar{D}_h$	$\bar{\sigma}$	$\bar{D}_h$	$\bar{\sigma}$
$CHCl_3$	1	2.03	0.432	3.85	0.835
	2	1.94	0.4	3.96	0.873
	3	1.93	0.38	2.4	0.42
DCM	1	1508.3	575	3.4	0.73
	2	5703	3081	2.5	0.54
	3	2.1	0.4	2.34	0.469
Toluene	1	2853	528	3.4	0.91
	2	4094	670	3.8	0.94
	3	2251	626	2.5	0.24
THF	1	0.75	0.17	1.3	0.32
	2	1.1	0.22	0.81	0.25
	3	0.98	0.28	1.3	0.33

1: Immediately after dissolving without THA-Br. 2: long-term measurement. 3: long-term measurement with THA-Br.

vi. Absorption and Fluorescence Emission Spectra

1. Free [Ru(dmbpy)$_3$]$^{2+}$ with Copolymers in Absence of Cages

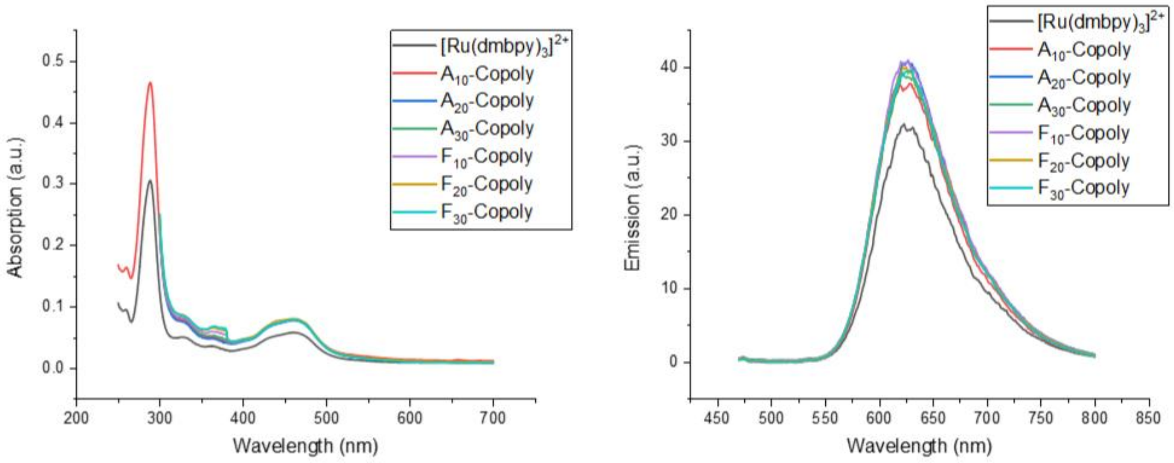

Figure S 1: Absorption (left) and fluorescence emission (right) spectra of free [Ru(dmbpy)$_3$]$^{2+}$ complex in chloroform in presence of 0.7 mol equivalent of the copolymers

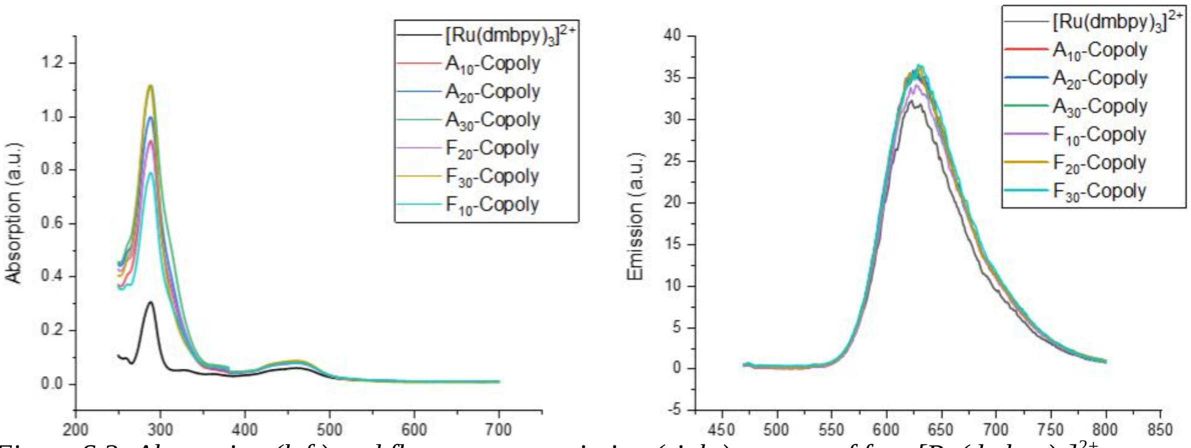

Figure S 2: Absorption (left) and fluorescence emission (right) spectra of free [Ru(dmbpy)$_3$]$^{2+}$ complex in chloroform in presence of 6.0 mol equivalent of the copolymers

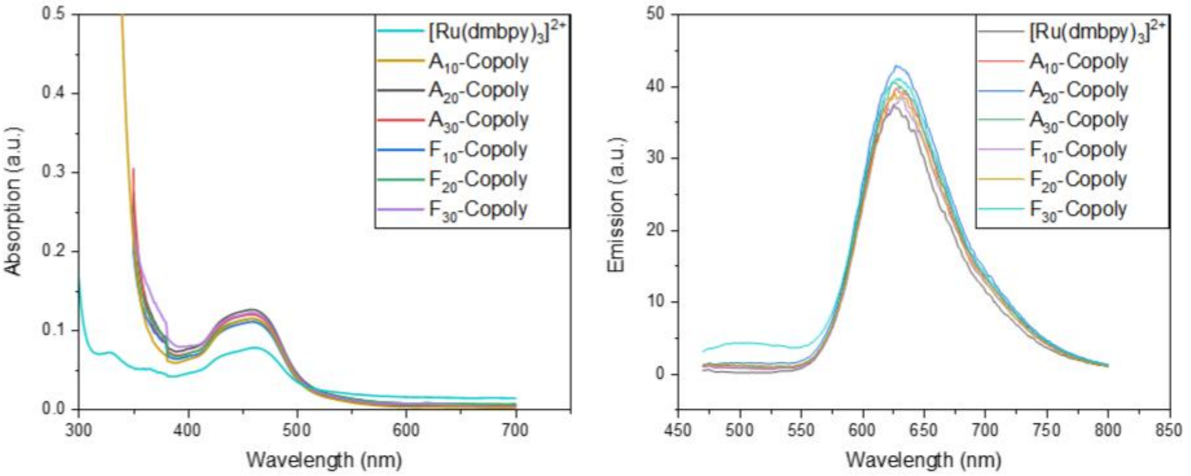

Figure S 3: Absorption (left) and fluorescence emission (right) spectra of free [Ru(dmbpy)$_3$]$^{2+}$ complex in chloroform in presence of 54.0 mol equivalent of the copolymers

2. **$[Ru(dmbpy)_3]^{2+}$** with Copolymers in Presence of **$[Rs_4]_6$**

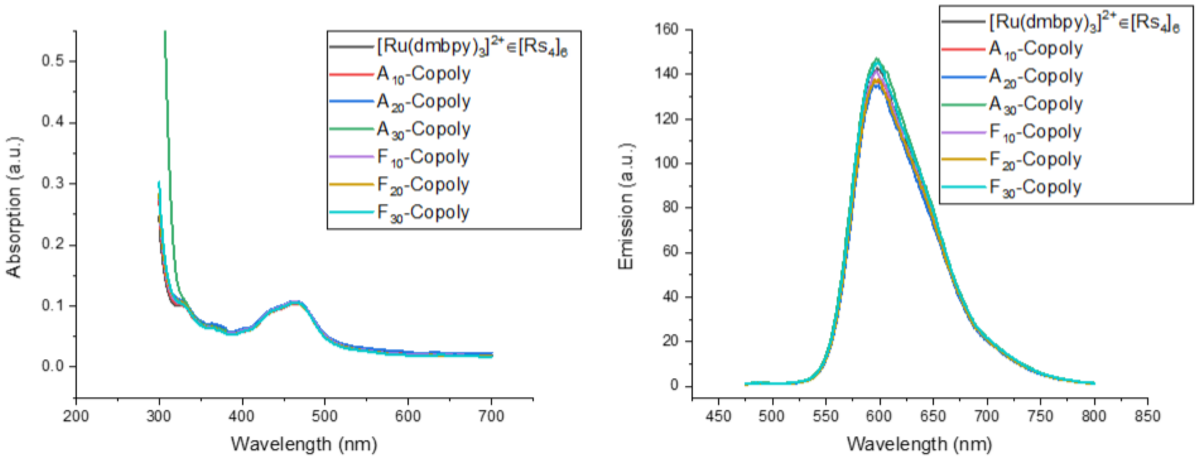

Figure S 4: Absorption (left) and fluorescence emission (right) spectra of encapsulated $[Ru(dmbpy)_3]^{2+}$ in $[Rs_4]_6$ ($[Ru(dmbpy)_3]^{2+} \epsilon [Rs_4]_6$) in presence of 0.7 mol equivalent of the copolymers

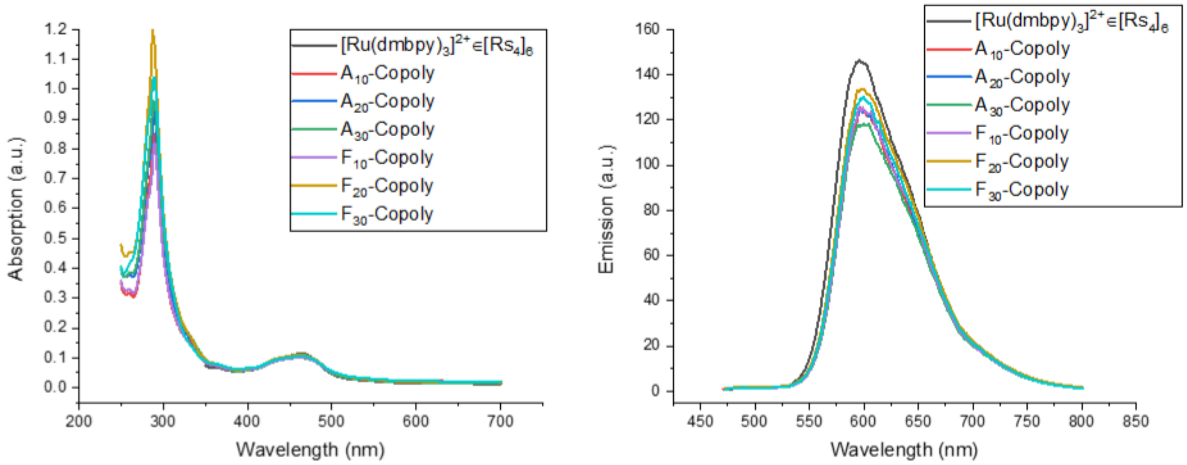

Figure S 5: Absorption (left) and fluorescence emission (right) spectra of encapsulated $[Ru(dmbpy)_3]^{2+}$ in $[Rs_4]_6$ ($[Ru(dmbpy)_3]^{2+} \epsilon [Rs_4]_6$) in presence of 6.0 mol equivalent of the copolymers

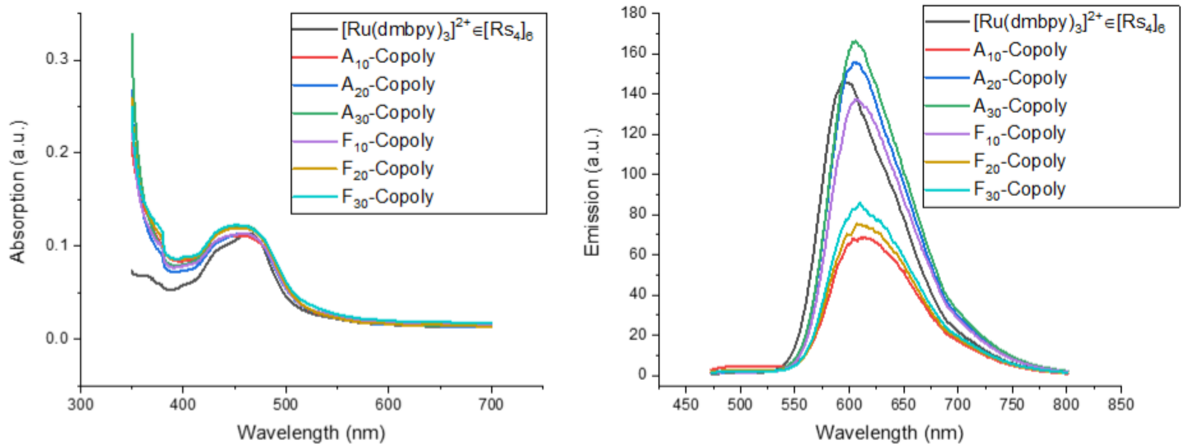

Figure S 6: Absorption (left) and fluorescence emission (right) spectra of encapsulated $[Ru(dmbpy)_3]^{2+}$ in $[Rs_4]_6$ ($[Ru(dmbpy)_3]^{2+} \epsilon [Rs_4]_6$) in presence of 54.0 mol equivalent of the copolymers.

3. **$[Ru(dmbpy)_3]^{2+}$** with Copolymers in Presence of **$[Rs_{11}]_6$**

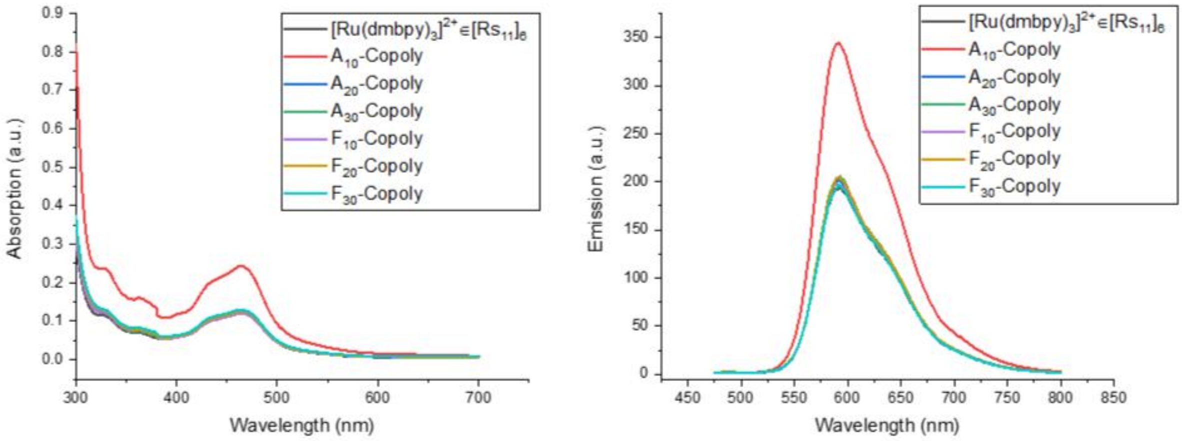

Figure S 7: Absorption (left) and fluorescence emission (right) spectra of encapsulated $[Ru(dmbpy)_3]^{2+}$ in $[Rs_{11}]_6$ ($[Ru(dmbpy)_3]^{2+} \epsilon [Rs_{11}]_6$) in presence of 0.7 mol equivalent of the copolymers

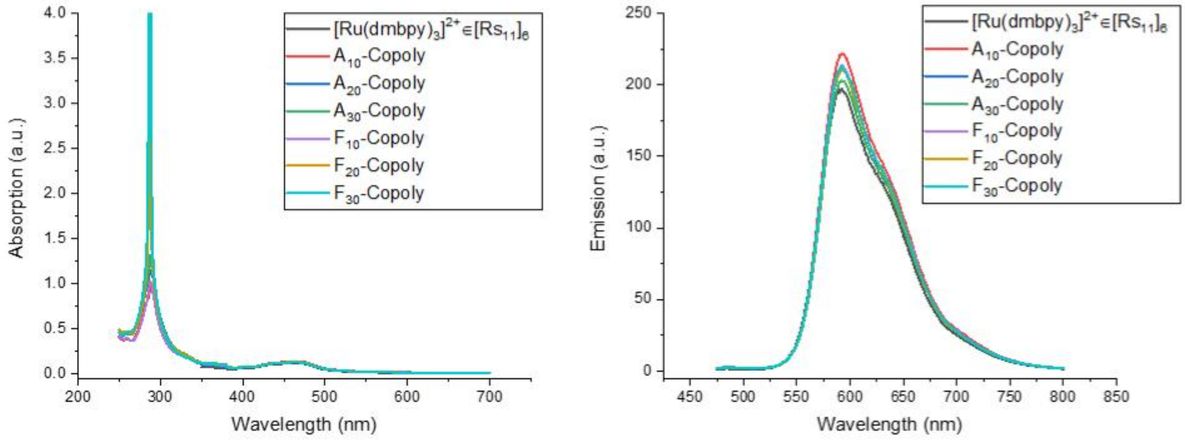

Figure S 8: Absorption (left) and fluorescence emission (right) spectra of encapsulated $[Ru(dmbpy)_3]^{2+}$ in $[Rs_{11}]_6$ ($[Ru(dmbpy)_3]^{2+} \epsilon [Rs_{11}]_6$) in presence of 6.0 mol equivalent of the copolymers

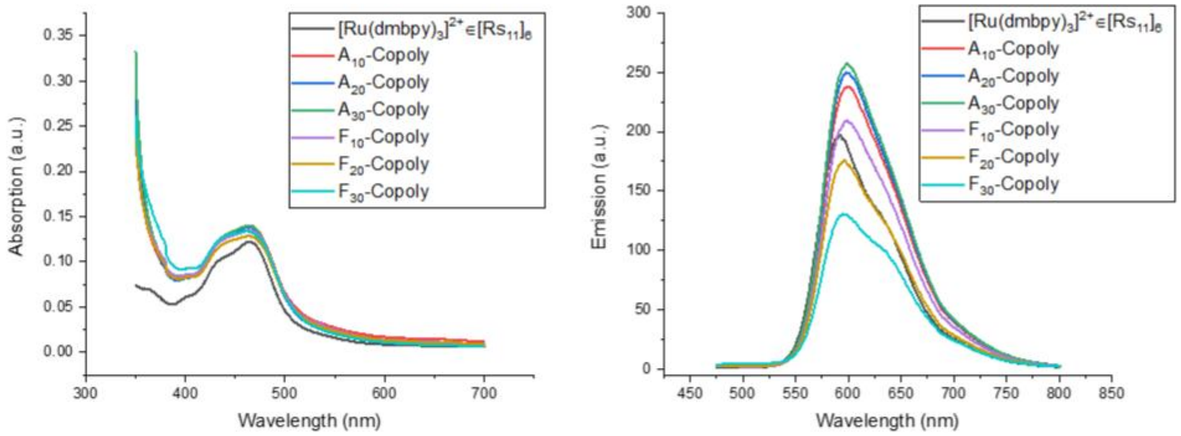

Figure S 9: Absorption (left) and fluorescence emission (right) spectra of encapsulated $[Ru(dmbpy)_3]^{2+}$ in $[Rs_{11}]_6$ ($[Ru(dmbpy)_3]^{2+} \epsilon [Rs_{11}]_6$) in presence of 54.0 mol equivalent of the copolymers

vii. 1D ^{1}H-NMR and ^{1}H-DOSY-NMR spectroscopy:

1. ^{1}H-NMR spectroscopy in Organic Solvents:

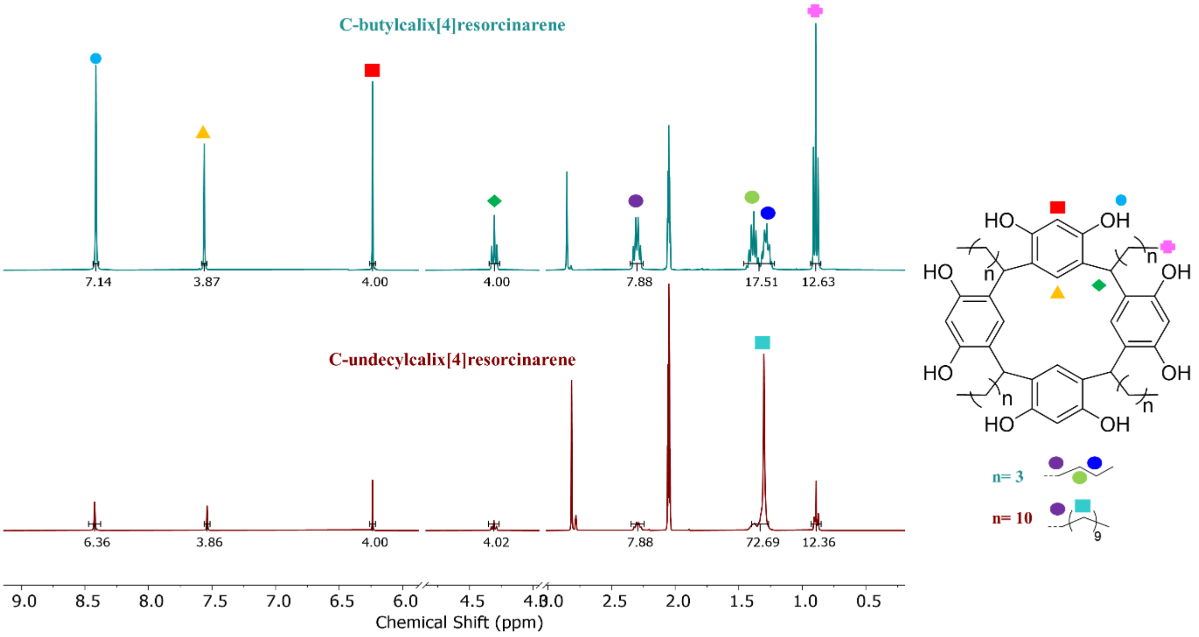

Figure S 10: ^{1}H-NMR spectra of $[Rs_4]_6$ (above) and $[Rs_{11}]_6$ (below) in acetone-d_6

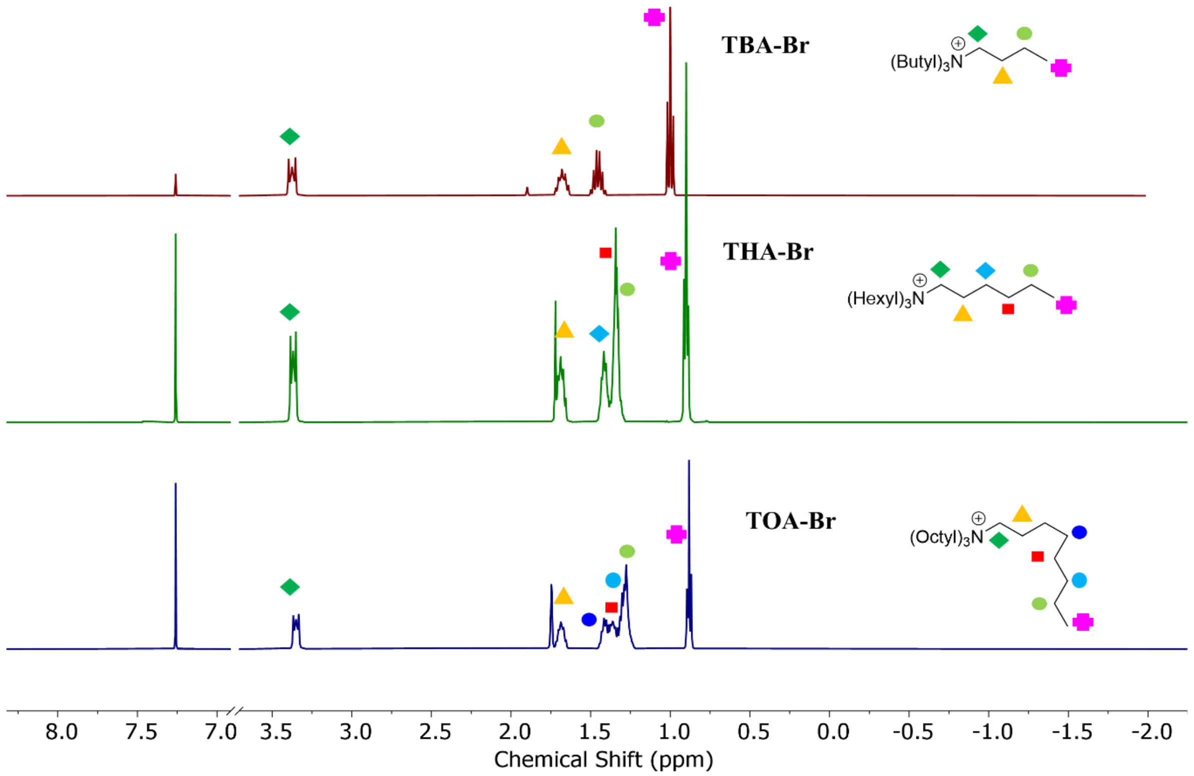

Figure S 11: ^{1}H-NMR spectra of TBA-Br, THA-Br and TOA-Br, respectively in $CDCl_3$

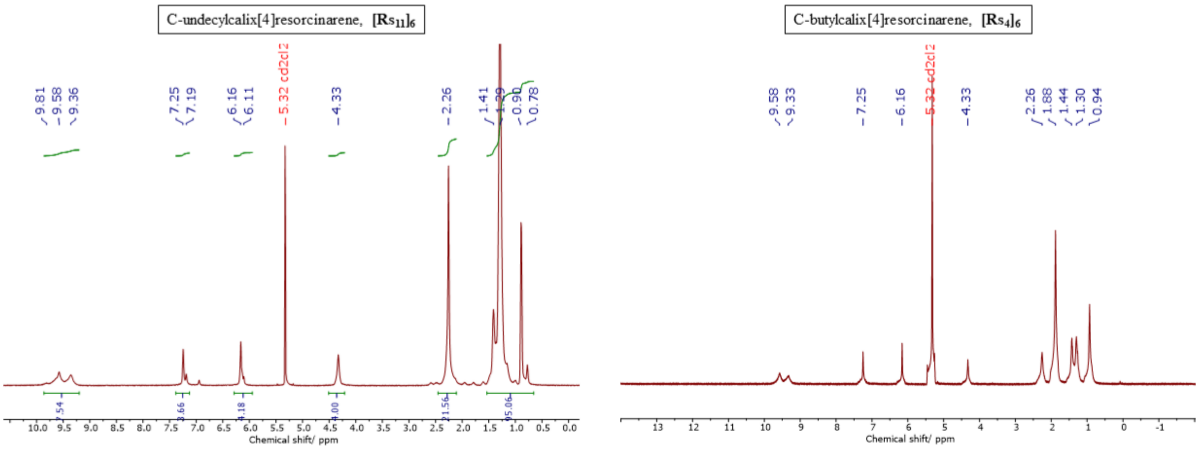

Figure S 12: ^{1}H-NMR spectra of [Rs$_4$]$_6$ (right) and [Rs$_{11}$]$_6$ (left) in CD_2Cl_2

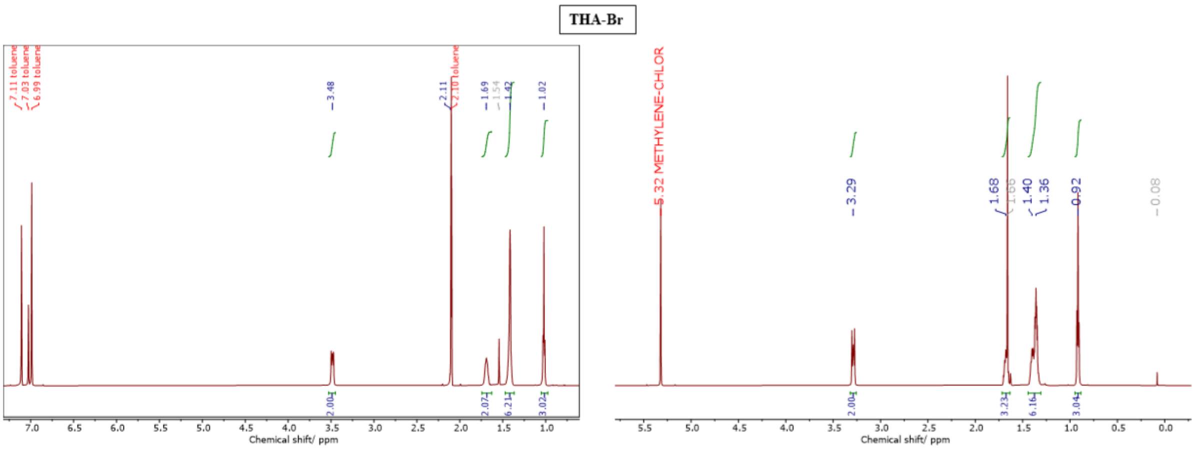

Figure S 13: ^{1}H-NMR spectra of THA-Br in toluene-d$_8$ (left) and in CD_2Cl_2 (right)

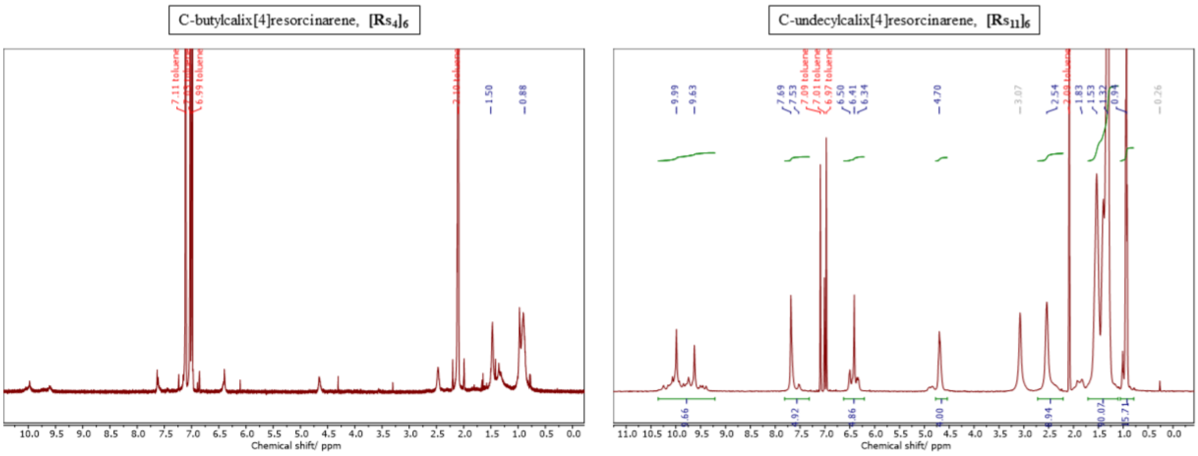

Figure S 14: ^{1}H-NMR spectra of [Rs$_4$]$_6$ (left) and [Rs$_{11}$]$_6$ (right) in toluene-d$_8$

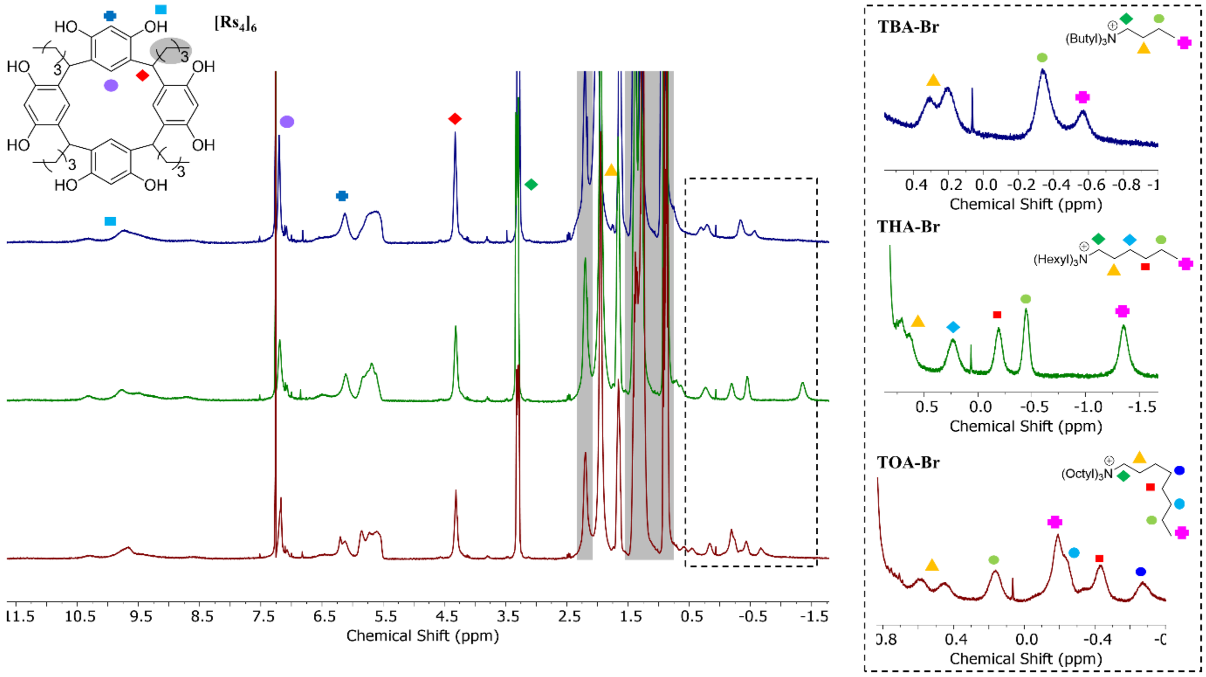

Figure S 15: ^{1}H-NMR spectra of TBA-Br, THA-Br and TOA-Br, respectively with $[Rs_4]_6$ in $CDCl_3$

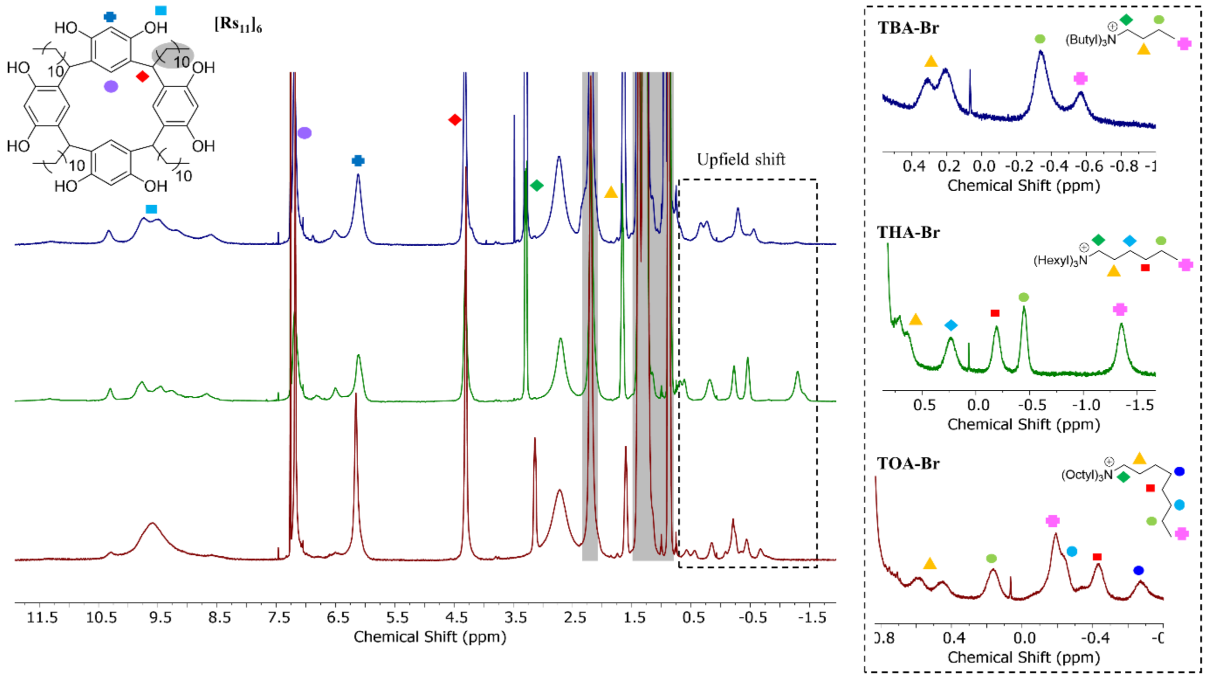

Figure S 16: ^{1}H-NMR spectra of TBA-Br, THA-Br and TOA-Br, respectively with $[Rs_{11}]_6$ in $CDCl_3$

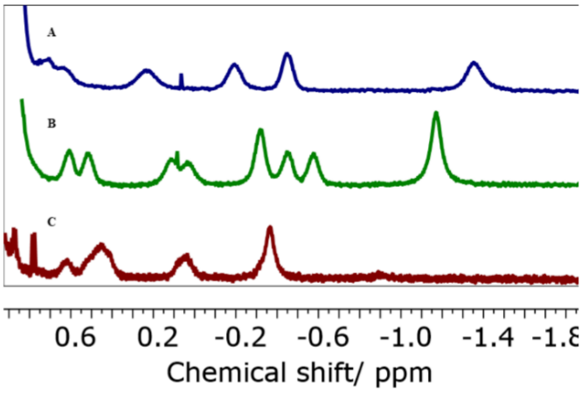

Figure S 17: ^{1}H-NMR spectra of encapsulated ***THA-Br*** *in* $[Rs_4]_6$ *cage in* $CDCl_3$ *(A), in DCM-*d_2 *(B), and in toluene-*d_8 *(C), showing the upfield shifts of the alkyl chains*

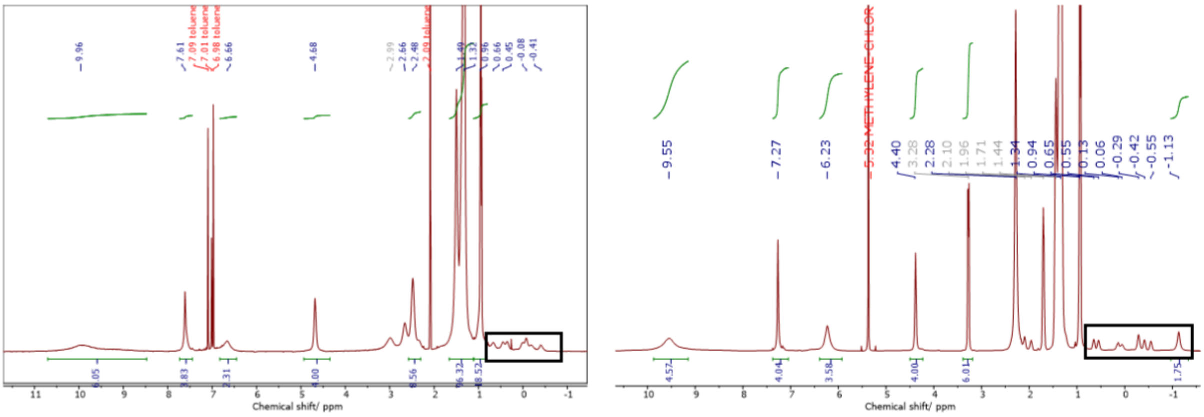

Figure S 18:Full ^{1}H-NMR spectra of encapsulated ***THA-Br*** *in* $[Rs_{11}]_6$ *cage in DCM-*d_2 *(right), and in toluene-*d_8 *(left), showing the upfield shifts of the alkyl chains*

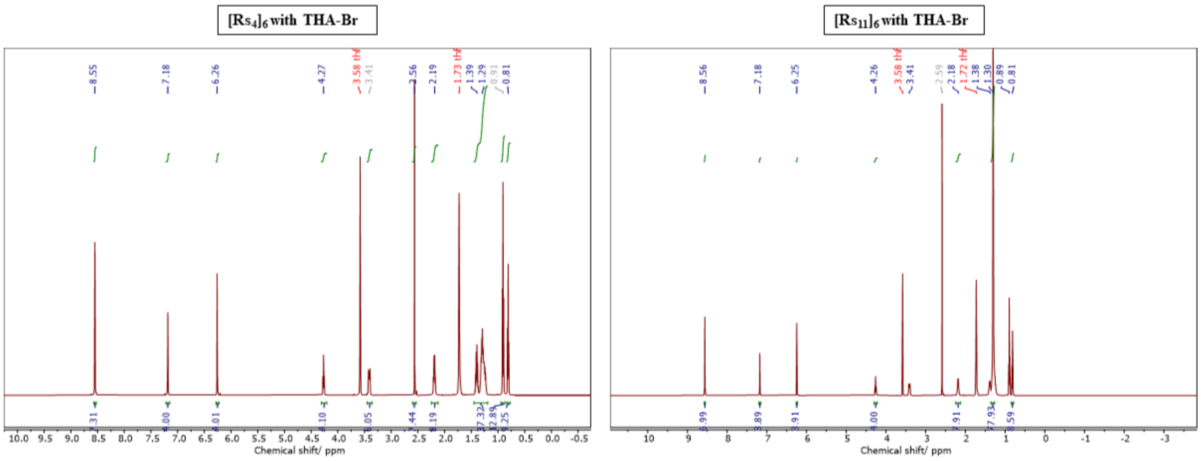

Figure S 19: ^{1}H-NMR spectra of $[Rs_4]_6$ *(left) and* $[Rs_{11}]_6$ *(right) with THA-Br in THF-*d_8

2. ^{1}H-DOSY-NMR spectroscopy in Organic Solvents:

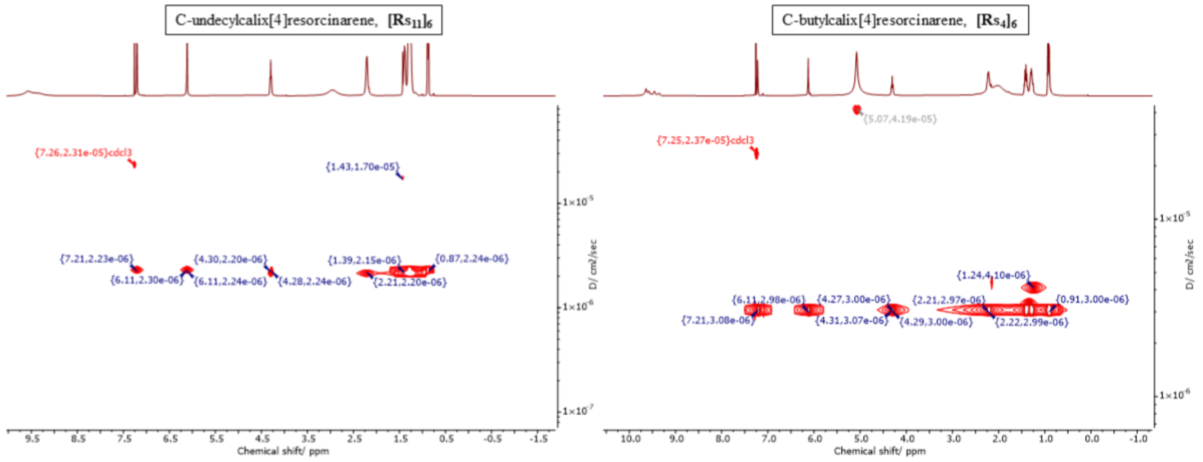

*Figure S 20: Fitted ^{1}H-DOSY NMR spectra of $[Rs_4]_6$ cage (right) and $[Rs_{11}]_6$ (left) showing diffusion coefficients (**D**) of the detected proton resonances in $CDCl_3$*

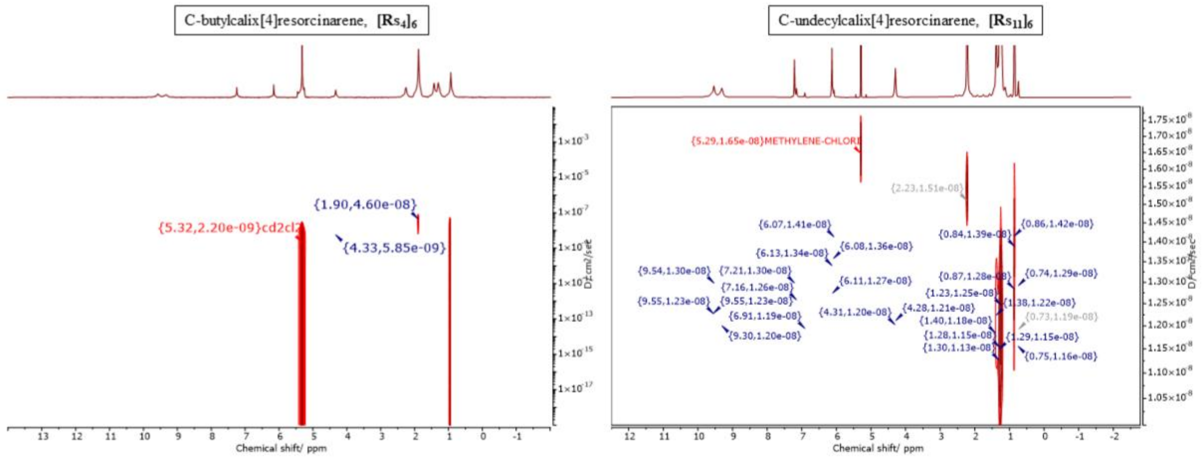

*Figure S 21: Fitted ^{1}H-DOSY NMR spectra of $[Rs_4]_6$ cage (left) and $[Rs_{11}]_6$ (right) showing diffusion coefficients (**D**) of the detected proton resonances in CD_2Cl_2*

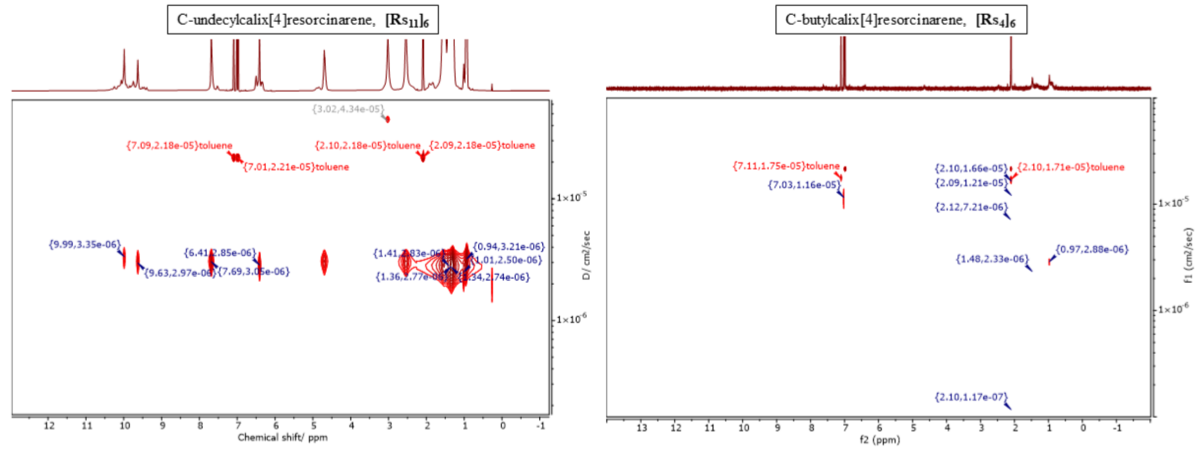

*Figure S 22: Fitted ^{1}H-DOSY NMR spectra of $[Rs_4]_6$ cage (right) and $[Rs_{11}]_6$ (left) showing diffusion coefficients (**D**) of the detected proton resonances in toluene-d_8*

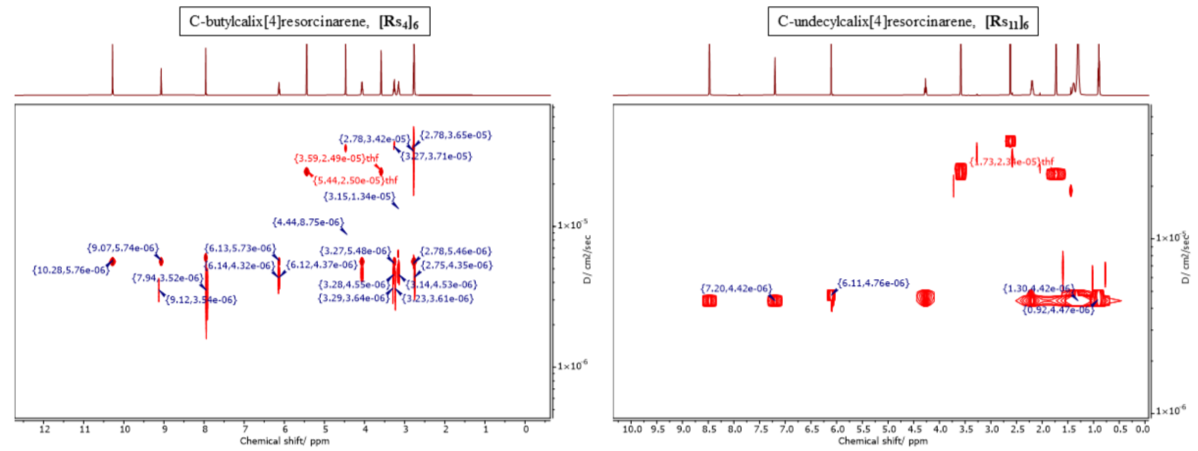

*Figure S 23: Fitted ^{1}H-DOSY NMR spectra of $[Rs_4]_6$ cage (left) and $[Rs_{11}]_6$ (right) showing diffusion coefficients (**D**) of the detected proton resonances in THF-d_8*

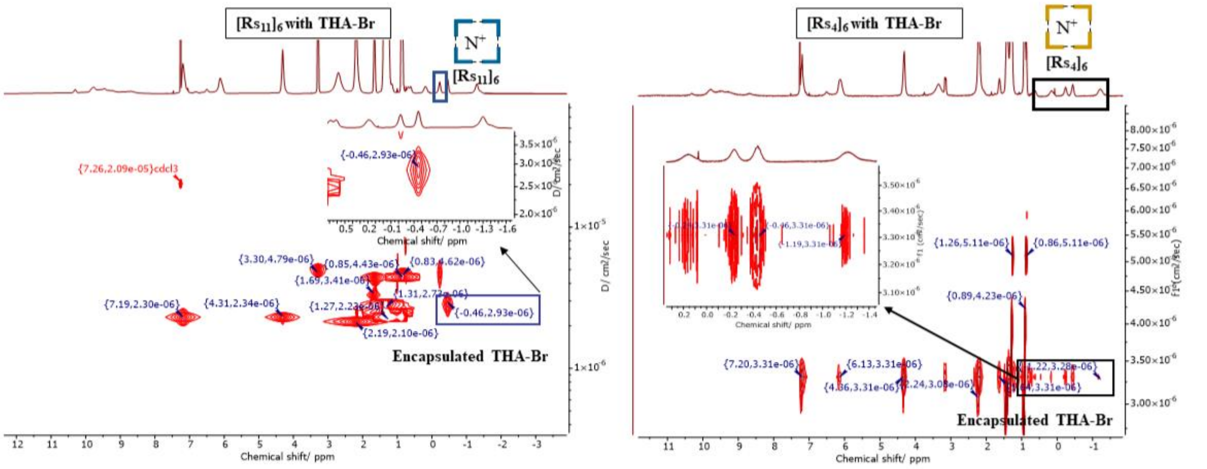

*Figure S 24: Fitted ^{1}H-DOSY NMR spectra of $[Rs_4]_6$ cage (right) and $[Rs_{11}]_6$ (left) with THA-Br showing diffusion coefficients (**D**) of the detected proton resonances in $CDCl_3$*

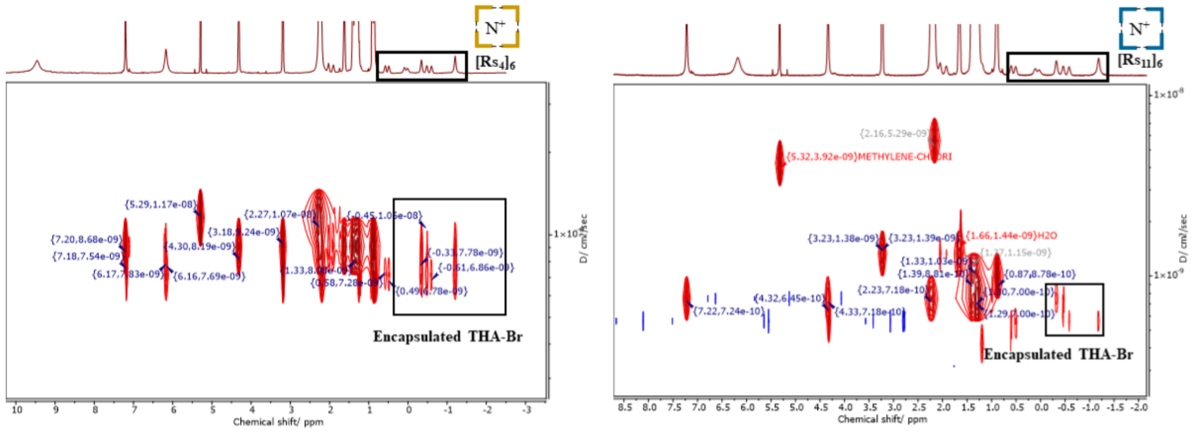

*Figure S 25: Fitted ^{1}H-DOSY NMR spectra of $[Rs_4]_6$ cage (left) and $[Rs_{11}]_6$ (right) with THA-Br showing diffusion coefficients (**D**) of the detected proton resonances in CD_2Cl_2*

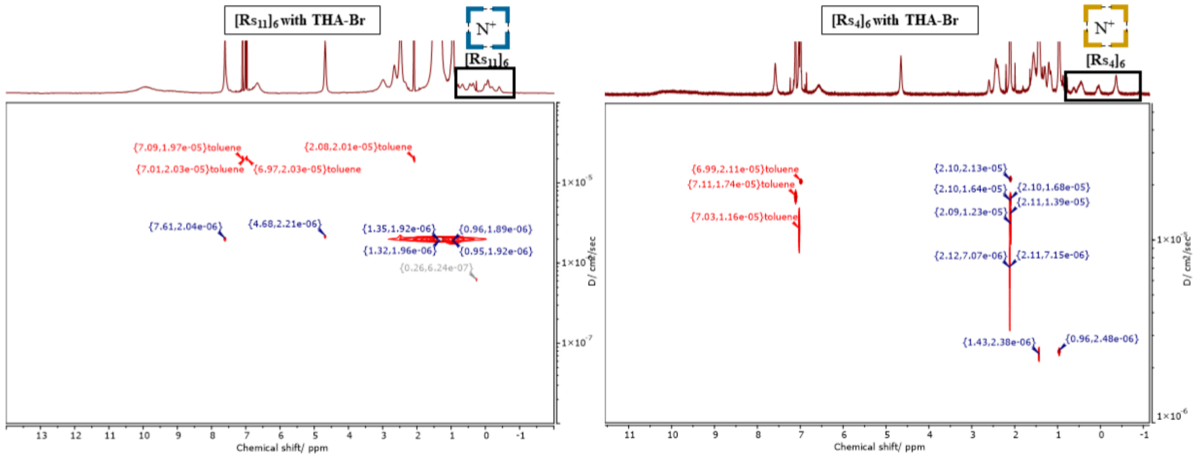

Figure S 26: Fitted [1]*H-DOSY NMR spectra of [Rs*$_4$*]*$_6$ *cage (right) and [Rs*$_{11}$*]*$_6$ *(left) with THA-Br showing diffusion coefficients (**D**) of the detected proton resonances in toluene-d*$_8$

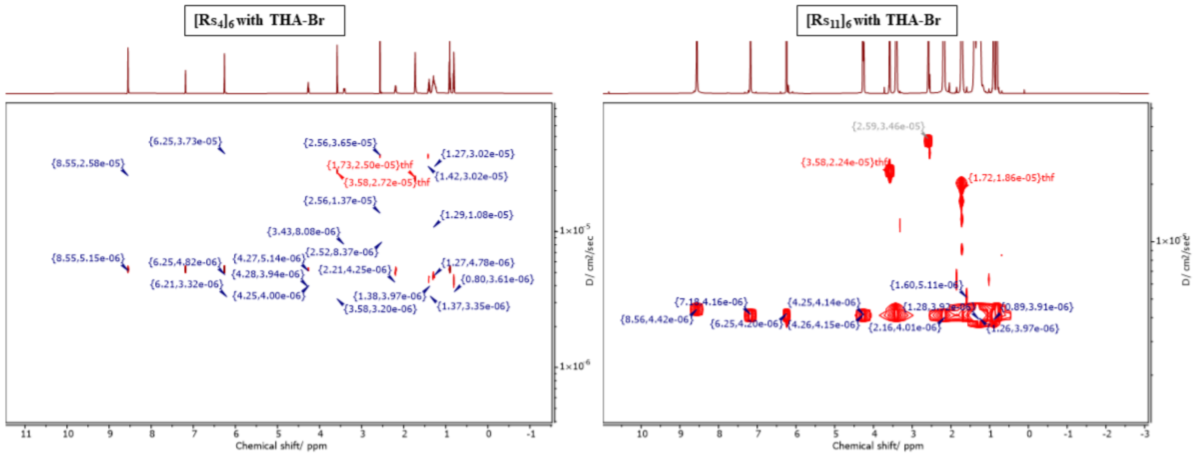

Figure S 27: Fitted [1]*H-DOSY NMR spectra of [Rs*$_4$*]*$_6$ *cage (left) and [Rs*$_{11}$*]*$_6$ *(right) with THA-Br showing diffusion coefficients (**D**) of the detected proton resonances in THF-d*$_8$

viii. Dynamic Light Scattering Measurements:

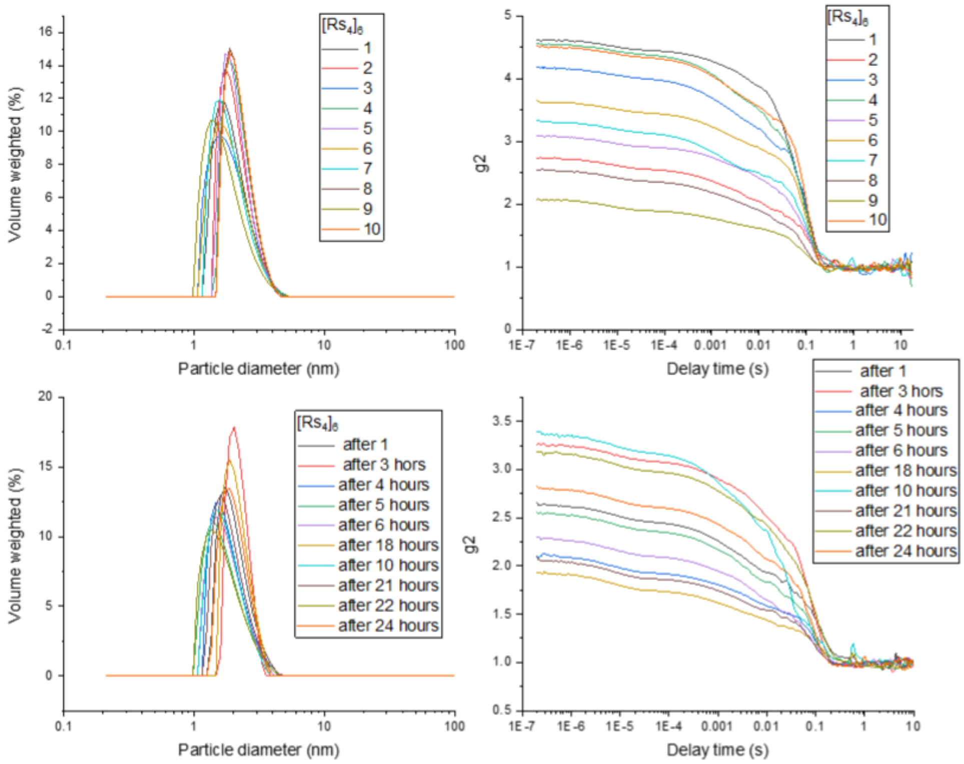

Figure S 28: Volume-weighted hydrodynamic radii of $[Rs_4]_6$ immediately after dissolving (above) and delayed (below) with the corresponding correlation functions in $CHCl_3$

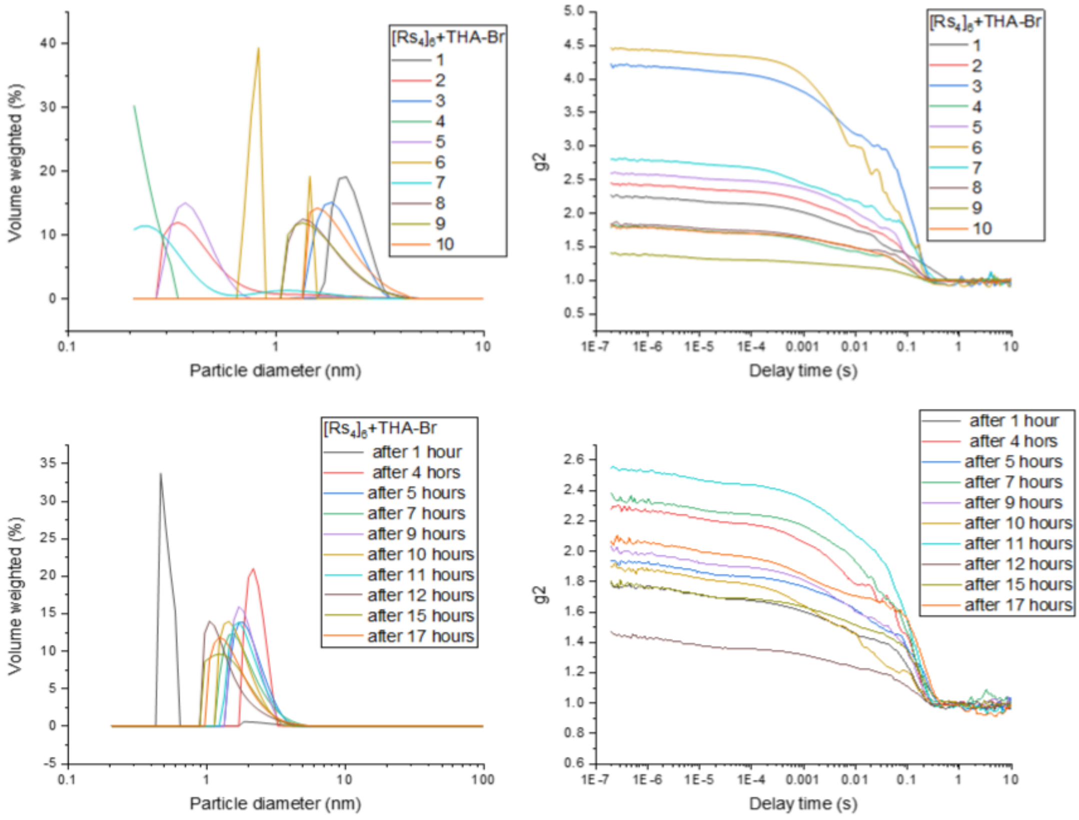

Figure S 29: Volume-weighted hydrodynamic radii of $[Rs_4]_6$ with THA-Br immediately after dissolving (above) and delayed (below) with the corresponding correlation functions in $CHCl_3$

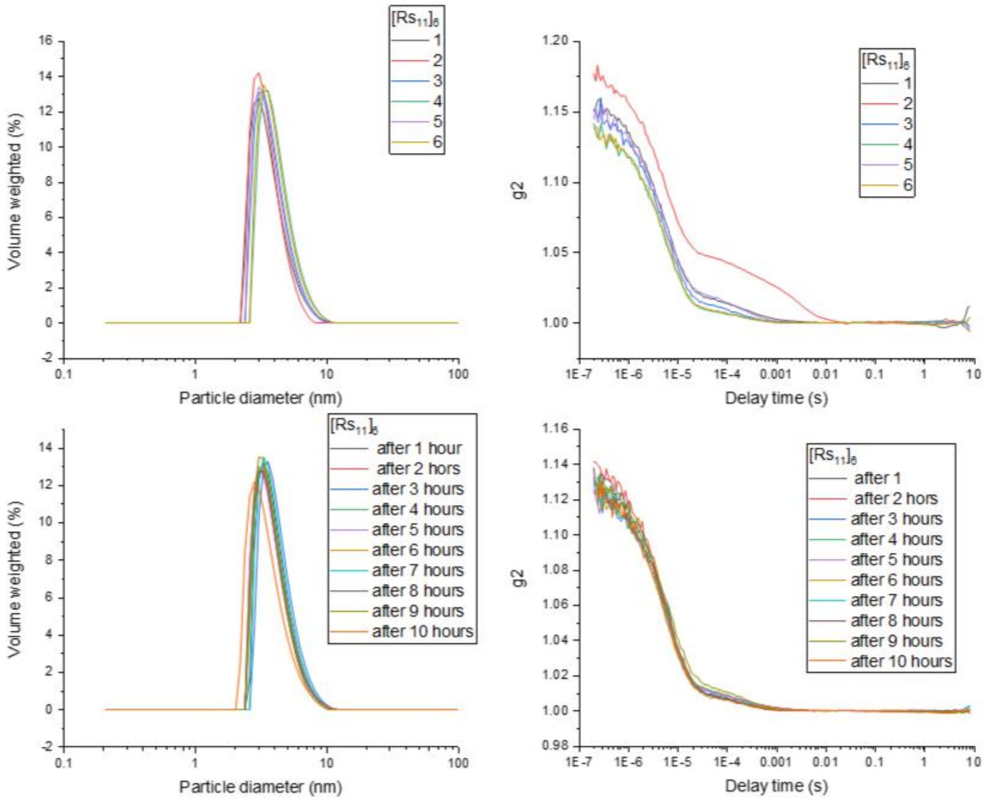

Figure S 30: Volume-weighted hydrodynamic radii of $[Rs_{11}]_6$ immediately after dissolving (above) and delayed (below) with the corresponding correlation functions in $CHCl_3$

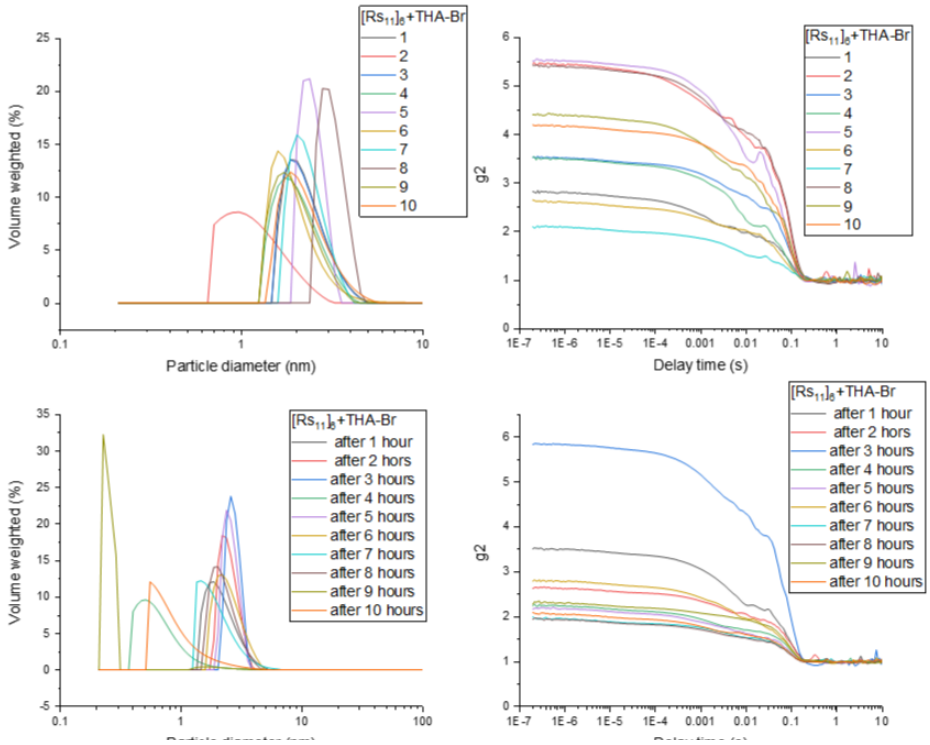

Figure S 31: Volume-weighted hydrodynamic radii of $[Rs_{11}]_6$ with THA-Br immediately after dissolving (above) and delayed (below) with the corresponding correlation functions in $CHCl_3$

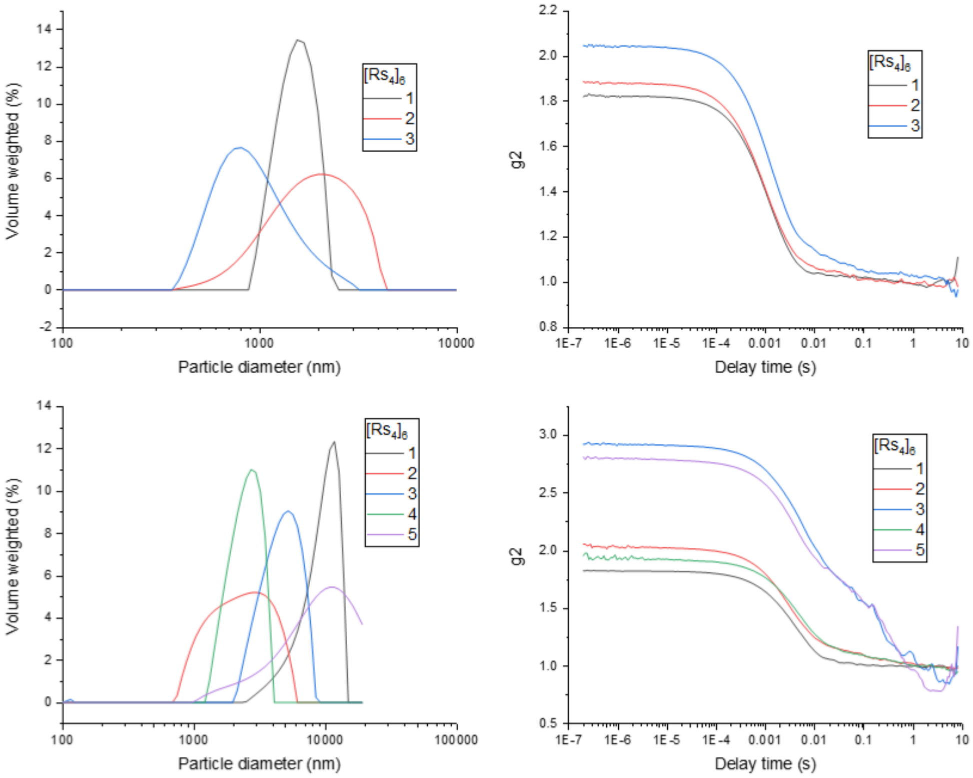

Figure S 32: Volume-weighted hydrodynamic radii of $[Rs_4]_6$ immediately after dissolving (above) and delayed (>2 days) (below) with the corresponding correlation functions in DCM

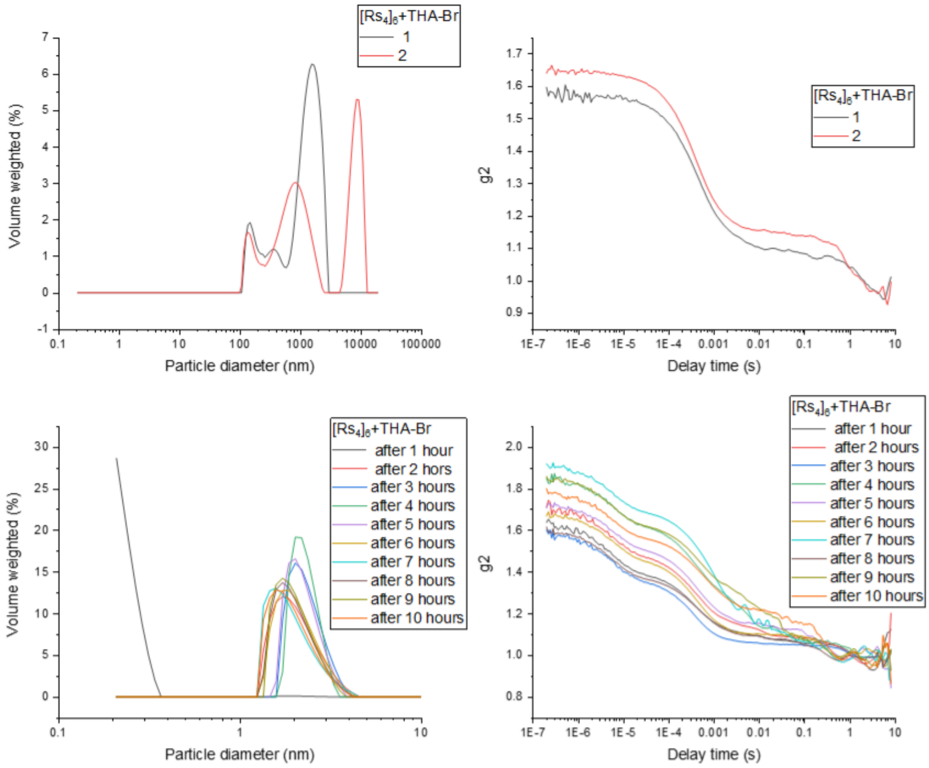

Figure S 33: Volume-weighted hydrodynamic radii of $[Rs_4]_6$ with THA-Br immediately after dissolving (above) and delayed (below) with the corresponding correlation functions in DCM

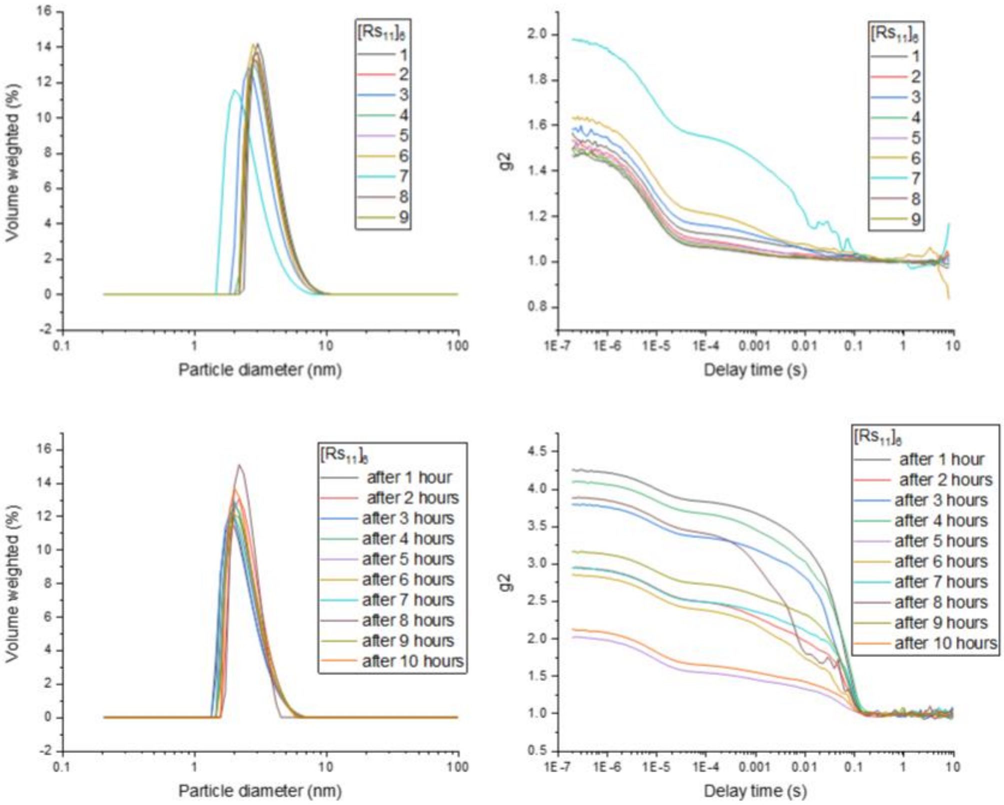

Figure S 34: Volume-weighted hydrodynamic radii of $[Rs_{11}]_6$ immediately after dissolving (above) and delayed (below) with the corresponding correlation functions in DCM

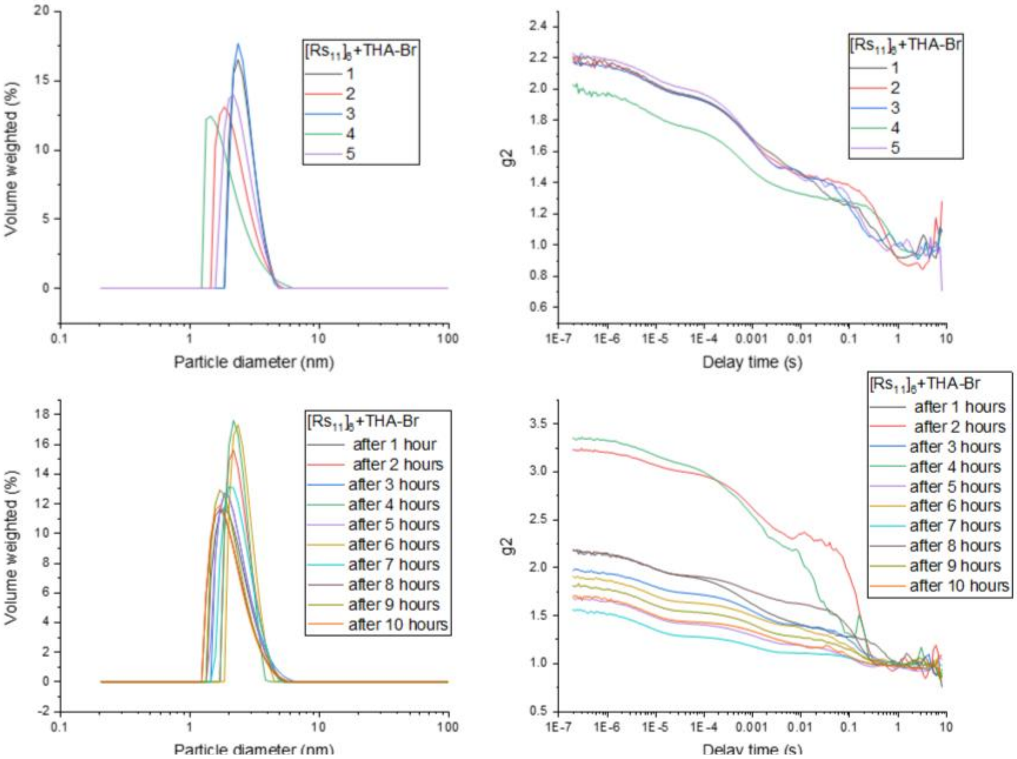

Figure S 35: Volume-weighted hydrodynamic radii of $[Rs_{11}]_6$ with THA-Br immediately after dissolving (above) and delayed (below) with the corresponding correlation functions in DCM

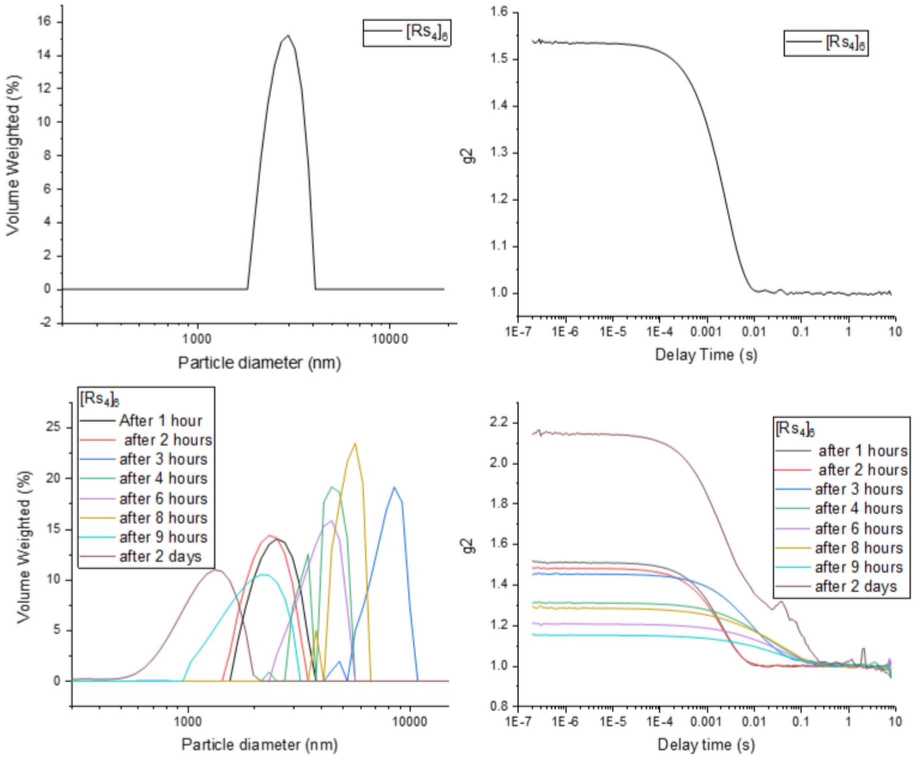

Figure S 36: Volume-weighted hydrodynamic radii of $[Rs_4]_6$ immediately after dissolving (above) and delayed (below) with the corresponding correlation functions in toluene

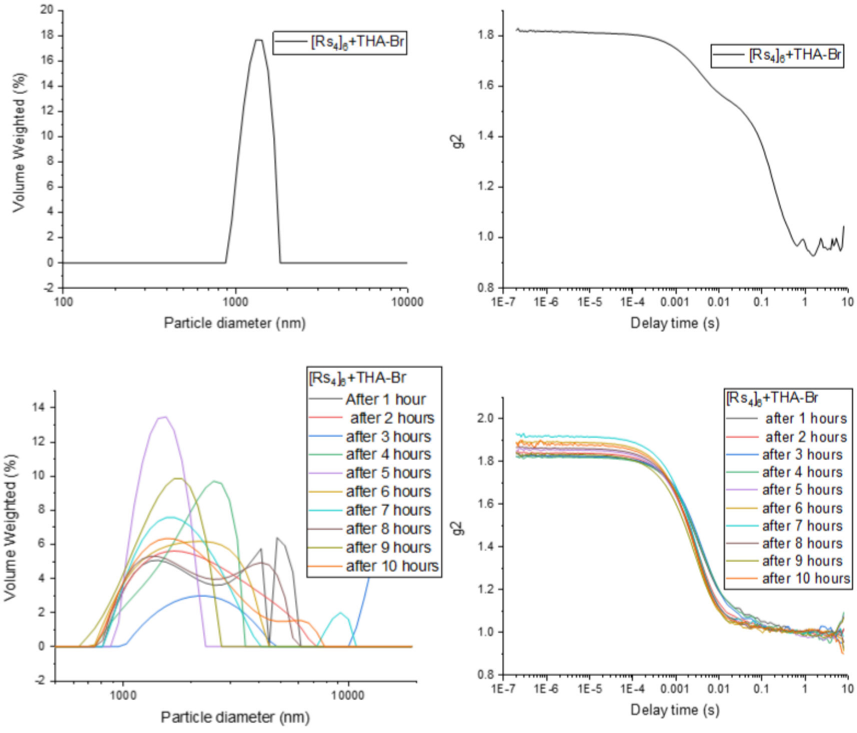

Figure S 37: Volume-weighted hydrodynamic radii of $[Rs_4]_6$ with THA-Br immediately after dissolving (above) and delayed (below) with the corresponding correlation functions in toluene

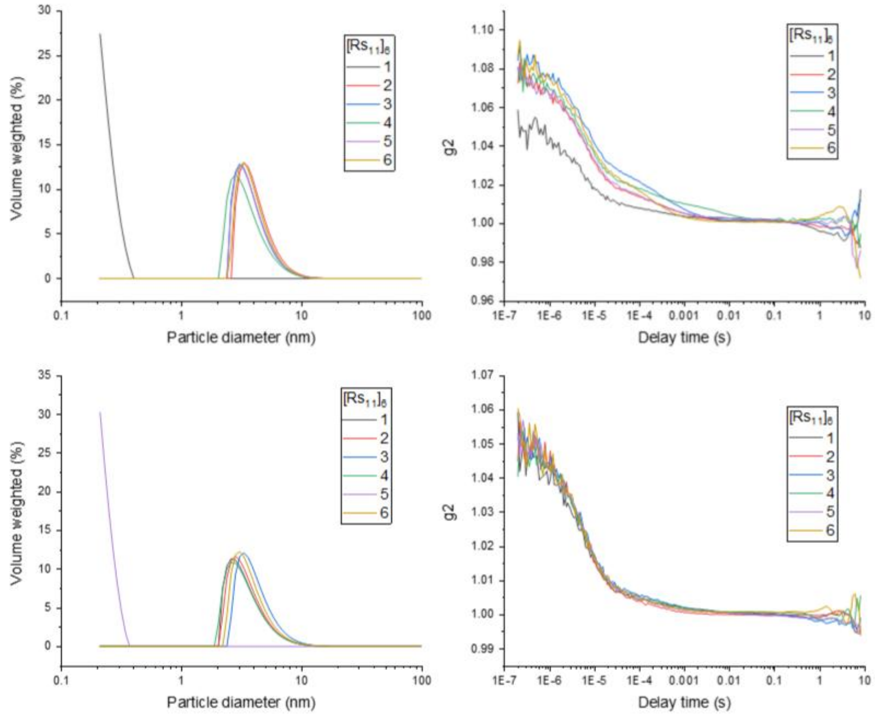

Figure S 38: Volume-weighted hydrodynamic radii of $[Rs_{11}]_6$ immediately after dissolving (above) and delayed (below) with the corresponding correlation functions in toluene

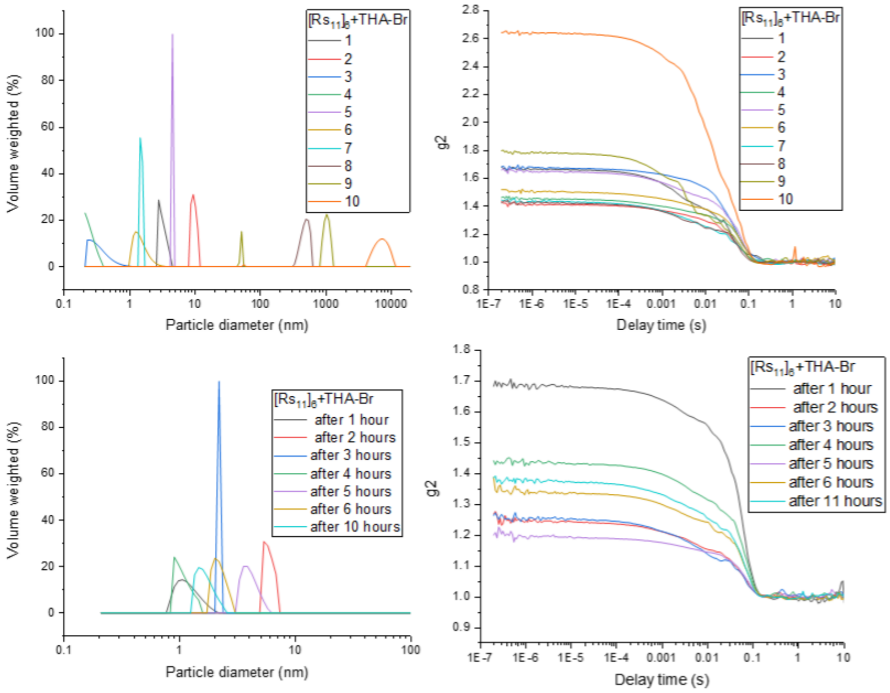

Figure S 39: Volume-weighted hydrodynamic radii of $[Rs_{11}]_6$ with THA-Br immediately after dissolving (above) and delayed (below) with the corresponding correlation functions in toluene

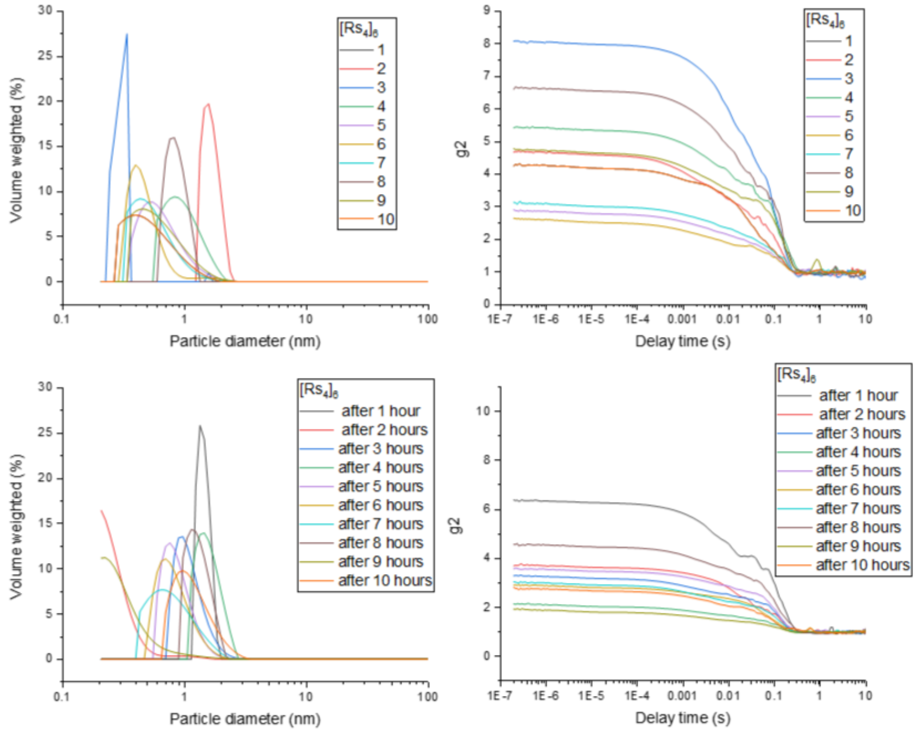

Figure S 40: Volume-weighted hydrodynamic radii of $[Rs_4]_6$ immediately after dissolving (above) and delayed (below) with the corresponding correlation functions in THF

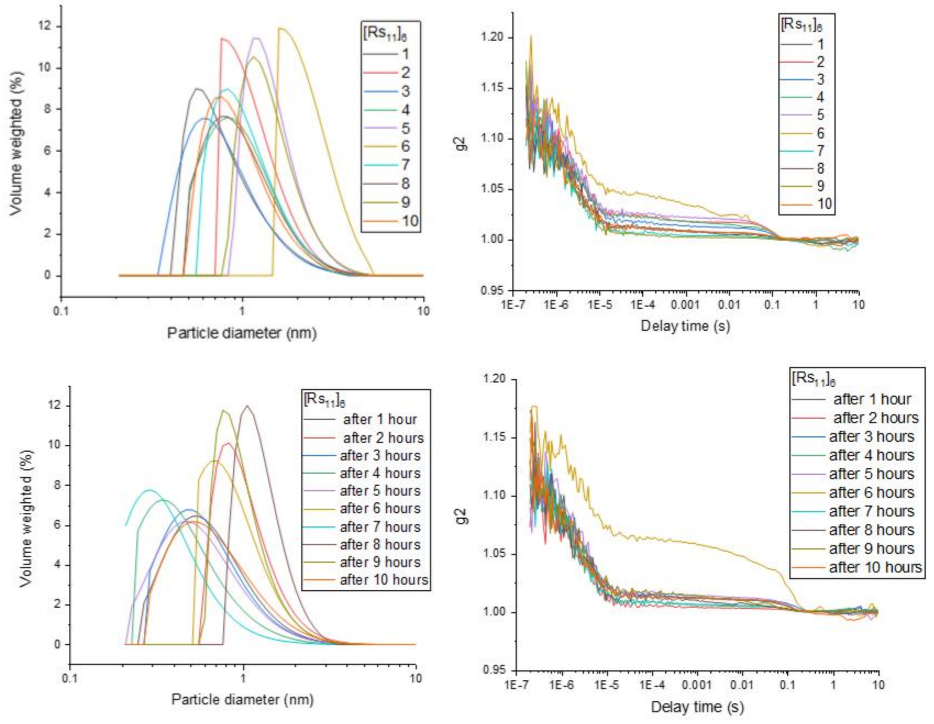

Figure S 41: Volume-weighted hydrodynamic radii of $[Rs_{11}]_6$ immediately after dissolving (above) and delayed (below) with the corresponding correlation functions in THF

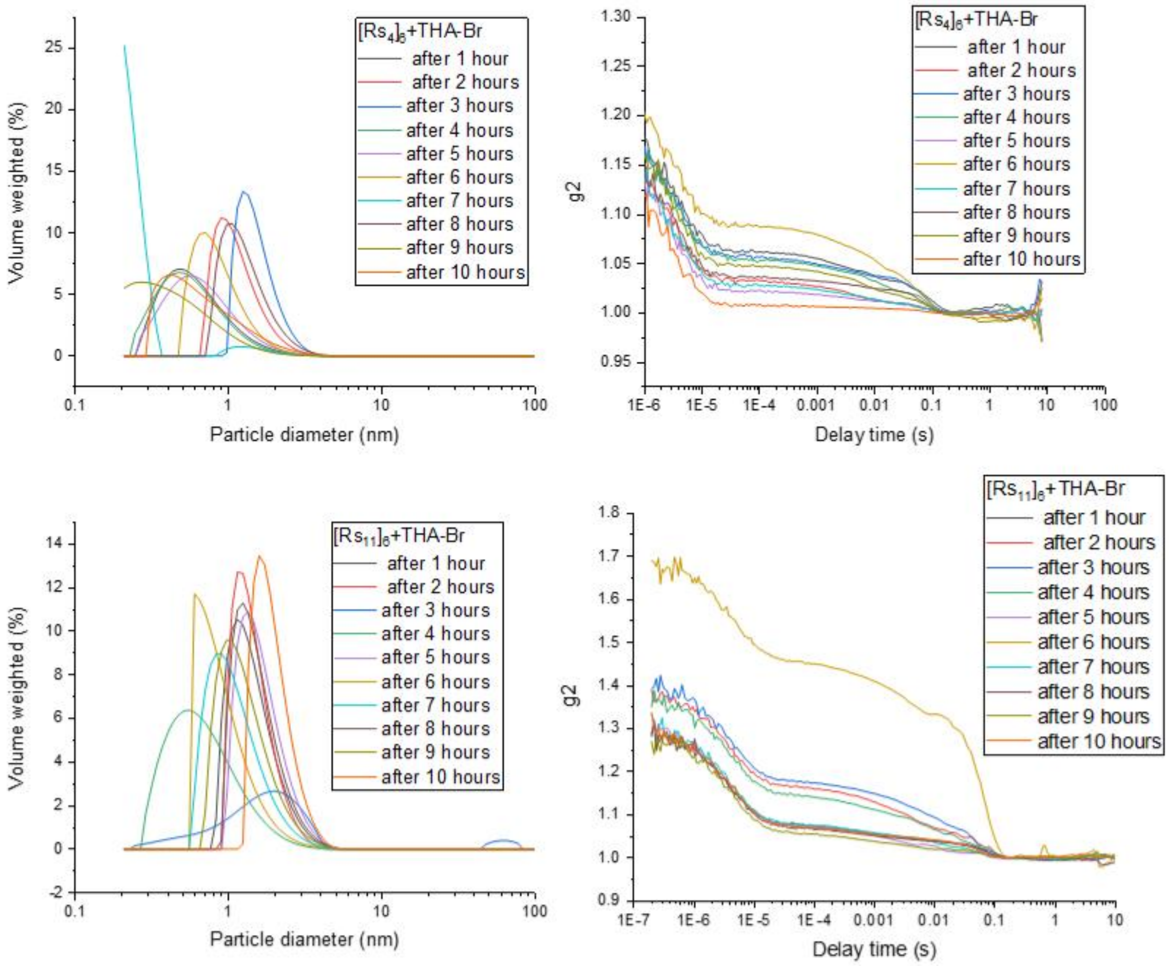

Figure S 42: Volume-weighted hydrodynamic radii of $[Rs_4]_6$ (above) and $[Rs_{11}]_6$ (below) with THA-Br and their corresponding correlation functions in THF

ix. ESI-ToF Mass Spectroscopy:

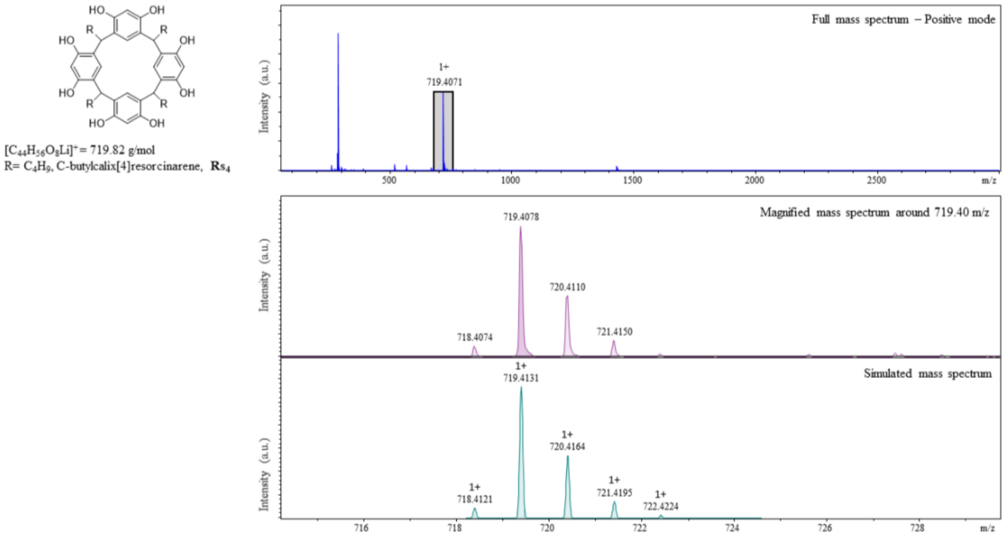

Figure S 43: Detected and simulated ESI-ToF mass spectrum of Rs_4 unit

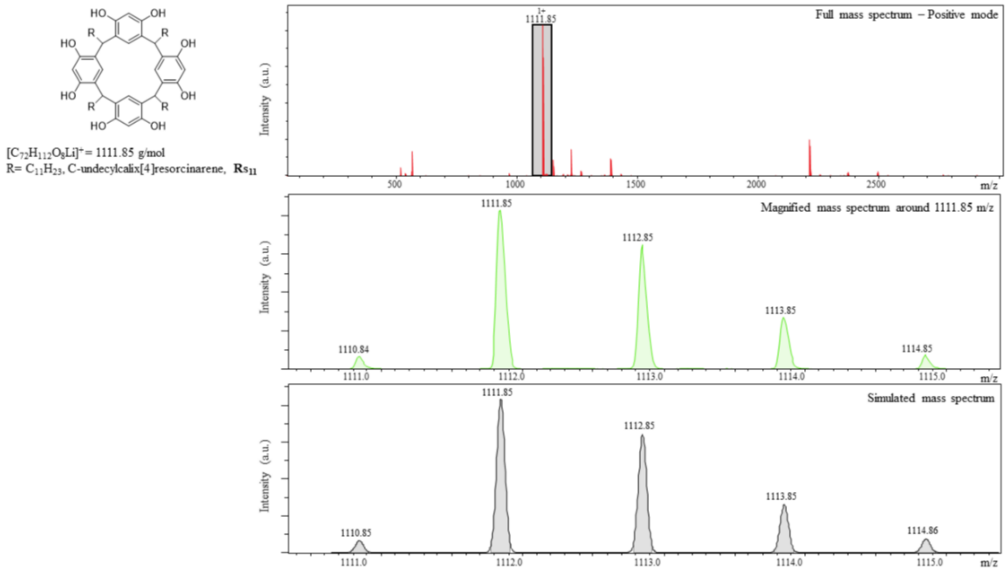

Figure S 44: Detected and simulated ESI-ToF mass spectrum of Rs_{11} unit

III. References

1. M. Chwastek, P. Cmoch and A. Szumna, *Journal of the American Chemical Society*, 2022, **144**, 5350-5358.

2. I. Elidrisi, S. Negin, P. V. Bhatt, T. Govender, H. G. Kruger, G. W. Gokel and G. E. M. Maguire, *Organic & Biomolecular Chemistry*, 2011, **9**, 4498-4506.
3. J. B. Niederl and H. J. Vogel, *Journal of the American Chemical Society*, 1940, **62**, 2512-2514.
